# TMEM16F Expressed in Kupffer Cells Regulates Liver Inflammation and Metabolism to Protect Against *Listeria Monocytogenes*

**DOI:** 10.1002/advs.202402693

**Published:** 2024-08-13

**Authors:** Jianlong Tang, Hua Song, Shimin Li, Sin Man Lam, Jieming Ping, Mengyun Yang, Na Li, Teding Chang, Ze Yu, Weixiang Liu, Yan Lu, Min Zhu, Zhaohui Tang, Zheng Liu, Yusong R. Guo, Guanghou Shui, André Veillette, Zhutian Zeng, Ning Wu

**Affiliations:** ^1^ Department of Immunology School of Basic Medicine Tongji Medical College Huazhong University of Science and Technology (HUST) Wuhan 430030 China; ^2^ The First Affiliated Hospital of Anhui Medical University and Institute of Clinical Immunology Anhui Medical University Hefei 230032 China; ^3^ The CAS Key Laboratory of Innate Immunity and Chronic Disease School of Basic Medical Sciences Division of Life Sciences and Medicine University of Science and Technology of China Hefei 230001 China; ^4^ State Key Laboratory of Molecular Developmental Biology Institute of Genetics and Developmental Biology Chinese Academy of Sciences Beijing 100101 China; ^5^ University of Chinese Academy of Sciences Beijing 100101 China; ^6^ Department of biochemistry and molecular biology School of Basic Medicine Tongji Medical College Huazhong University of Science and Technology Wuhan 430030 China; ^7^ Department of Traumatic Surgery Tongji Trauma Center Tongji Hospital Tongji Medical College Huazhong University of Science and Technology Wuhan 430030 China; ^8^ Department of Otolaryngology‐Head and Neck Surgery Tongji Hospital Tongji Medical College Huazhong University of Science and Technology No. 1095 Jiefang Avenue Wuhan 430030 China; ^9^ Department of Clinical Immunology The Third Affiliated Hospital of Sun Yat‐sen University Guangzhou 510630 China; ^10^ Department of Thoracic Surgery Tongji Hospital Tongji Medical College Huazhong University of Science and Technology Wuhan 430030 China; ^11^ Cell Architecture Research Center Tongji Medical College Huazhong University of Science and Technology Wuhan 430030 China; ^12^ Laboratory of Molecular Oncology Institut de recherches cliniques de Montréal (IRCM) Montréal Québec H2W1R7 Canada; ^13^ Department of Medicine University of Montréal Montréal Québec H3T 1J4 Canada; ^14^ Department of Medicine McGill University Montréal Québec H3G 1Y6 Canada; ^15^ Department of Oncology The First Affiliated Hospital of USTC University of Science and Technology of China Hefei 230001 China

**Keywords:** inflammation, kupffer cell, lipid scrambling, *listeria monocytogenes*, macrophage, plasma membrane integrity, TMEM16F

## Abstract

Infection by bacteria leads to tissue damage and inflammation, which need to be tightly controlled by host mechanisms to avoid deleterious consequences. It is previously reported that TMEM16F, a calcium‐activated lipid scramblase expressed in various immune cell types including T cells and neutrophils, is critical for the control of infection by bacterium *Listeria monocytogenes* (*Lm*) in vivo. This function correlated with the capacity of TMEM16F to repair the plasma membrane (PM) damage induced in T cells in vitro, by the *Lm* toxin listeriolysin O (LLO). However, whether the protective effect of TMEM16F on *Lm* infection in vivo is mediated by an impact in T cells, or in other cell types, is not determined. Herein, the immune cell types and mechanisms implicated in the protective effect of TMEM16F against *Lm* in vivo are elucidated. Cellular protective effects of TMEM16F correlated with its capacity of lipid scrambling and augment PM fluidity. Using cell type‐specific TMEM16F‐deficient mice, the indication is obtained that TMEM16F expressed in liver Kupffer cells (KCs), but not in T cells or B cells, is key for protection against *Listeria* in vivo. In the absence of TMEM16F, *Listeria* induced PM rupture and fragmentation of KCs in vivo. KC death associated with greater liver damage, inflammatory changes, and dysregulated liver metabolism. Overall, the results uncovered that TMEM16F expressed in Kupffer cells is crucial to protect the host against *Listeria* infection. This influence is associated with the capacity of Kupffer cell‐expressed TMEM16F to prevent excessive inflammation and abnormal liver metabolism.

## Graphical abstract

Bacterial infection causes tissue damage and inflammation, requiring control. TMEM16F, a Ca^2+^‐activated lipid scramblase, is vital for controlling *Listeria monocytogenes* infection. This study shows TMEM16F in liver‐resident macrophages, Kupffer cells, prevents plasma membrane injury and cell death by scrambling plasma membrane lipids, thereby reducing liver damage and inflammation. Thus, Kupffer cell‐expressed TMEM16F is crucial for protection against *Listeria*.

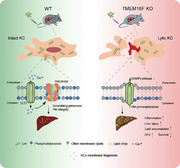

## 1 Introduction

Bacterial pathogens infection is a leading cause of death globally. The priority of public health is to alleviate the burden of death due to the infection.^[^ ^1^ ^]^ *Listeria* is the second death‐threatening food‐borne bacterial pathogen, with ≈15 000 death estimated per year. *Listeria monocytogenes* (*Lm*) is a well‐defined model microorganism for the study of host–bacteria interactions and PM repair mechanisms.^[^ ^2^, ^3^ ^]^ As a facultative intracellular bacterial pathogen, *Listeria* enters the host cell via Internalin A and B (InlA and InlB).^[^ ^4^, ^5^ ^]^ Then, it escapes the phagocytic vacuole, to exploit the host actin system and eventually spread to neighboring cells.^[^ ^6^, ^7^ ^]^ *Lm* usually enters through the gastrointestinal tract, and disseminates via the blood to distal sites, causing systemic infection.^[^ ^8^ ^]^ *Listeria* employs various mechanisms to evade immune surveillance.^[^ ^9^, ^10^ ^]^ For instance, once entering the circulation, *Lm* is rapidly captured by liver‐resident macrophages, also known as Kupffer cells (KCs).^[^ ^11^ ^]^ *Lm* subsequently induces death of Kupffer cells, and triggers severe inflammation and tissue damage.^[^ ^12^, ^13^ ^]^

Microorganisms often injure cells by producing pore‐forming toxins (PFT) that damage the plasma membrane (PM), such as the Listeria‐associated listeriolysin O (LLO).^[^ ^14^, ^15^, ^16^ ^]^ Wounded PMs need to be healed instantly, and failure of PM repair can ultimately lead to cell death, and unleash the danger signals to fuel inflammation and cause immunopathology.^[^ ^17^, ^18^ ^]^ Eukaryotic cells have evolved various membrane repair mechanisms to protect themselves from this damage, by promptly sealing pores created in the PM.^[^ ^19^, ^20^, ^21^ ^]^ Small lesions are usually removed by endocytosis or the endosomal sorting complexes required for transport (ESCRT)‐mediated vesicle shedding, while large wounds are thought to be repaired by membrane patching.^[^ ^22^, ^23^, ^24^, ^25^, ^26^ ^]^

Among the *Listeria* virulence factors, LLO plays a significant role in pathogenicity.^[^ ^27^, ^28^, ^29^, ^30^ ^]^ LLO helps *Lm* to enter host cells and to escape from the vacuole, but it also wounds the PM by creating pores.^[^ ^28^, ^31^ ^]^ Unhealed PM in *Listeria* infection leads to cell death, inflammation, and innate immune responses.^[^ ^11^, ^12^, ^32^, ^33^, ^34^ ^]^ The *Listeria*‐containing membrane protrusions often displays externalized phosphatidylserine (PS), and efficient repair of LLO‐mediated membrane damage requires extracellular Ca^2+^, suggesting that a process of lipid scrambling happens at the wounded site on PM.^[^ ^7^ ^]^

Ca^2+^‐activated lipid scramblase TMEM16F (also known as anoctamin 6; ANO6) belongs to the TMEM16 family, consisting of ten paralogs with chloride channel and/or scramblase activities.^[^ ^35^, ^36^ ^]^ TMEM16F is involved in multiple physiological or pathologic processes, such as blood coagulation, bone mineralization, trophoblast fusion during embryo development, and syncytium formation during SARS‐CoV‐2 infection.^[^ ^30^, ^37^, ^38^, ^39^, ^40^, ^41^, ^42^, ^43^, ^44^, ^45^ ^]^ Loss of TMEM16F function in humans results in a blood coagulation disorder, named Scott syndrome, which is manifested by prolonged blood clotting time.^[^ ^38^, ^41^, ^45^ ^]^

Previously, we reported that TMEM16F was also critical for PM repair following pore formation, including in response to LLO, in cell types such as T cells and neutrophils, in vitro. TMEM16F was also needed to protect mice from the pathogenic effects of *Lm* in vivo. Lack of TMEM16F in all mouse cell types resulted in increased liver damage triggered by *Lm*. However, the precise cell type(s) and mechanism(s) responsible for this protective role were not established.

Here, we figured out both the cellular and molecular mechanism of TMEM16F in the protection of *Listeria* infection. We first uncovered that TMEM16F functions through scrambling lipid as a universal process, crucial for maintaining plasma membrane integrity in various primary cells and cell lines. Using cell type‐specific TMEM16F‐deficient mice, we identified that TMEM16F, particularly in Kupffer cells (KCs), serves as a safeguard against infection‐induced damage in the liver. Through intravital imaging, we observed the membrane rupture and fragmentation of Kupffer cells in the absence of TMEM16F during *Listeria* infection. We propose that lytic cell death resulted in the uncontrolled release of intracellular contents, intensifying inflammation, disrupting metabolism, and exacerbating tissue injury.

## 2 Results

### 2.1 Lipid Scrambling Essentially Contributes to the Resistance to PM Permeabilization Induced by *Lm*

**Figure 1:**
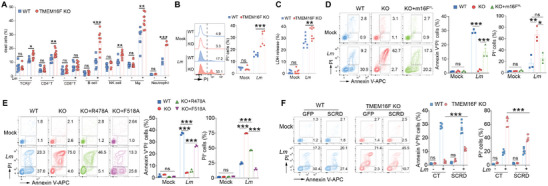
Lipid scrambling mediated by TMEM16F essentially counteracts cell death induced by *Lm*. A) Quantification of TCRβ^+^ T (TCRβ^+^NK1.1^−^) cell, CD4^+^ T (CD4^+^ CD8^−^) cell, CD8^+^ T (CD4^−^ CD8^+^) cell, B (B220^+^ TCRβ^−^) cell, NK (TCRβ^−^NK1.1^+^) cell, macrophage (F4/80^+^CD11b^+^) cell and neutrophil (Ly6G^+^CD11b^+^) cell permeabilization (DAPI^+^) in splenocytes infected by *Lm* for 1 h. The MOI of infected neutrophils was 50 and that of other immune cells was 5. (n = 3‐9). B) Plasma membrane damage in BMDM infected with *Lm* for 8h detected with PI staining (MOI = 15, n = 6). C) LDH release assay as in (B) (n = 8). D) Representative flow cytometry plots (left panel) of cells in WT, TMEM16F KO and KO rescued with full length TMEM16F (KO+m16F^FL^) RMA cells ± *Lm* for 2h and stained with Annexin V and PI. Quantification of PS exposure (Annexin V^+^PI^−^) and cell death (PI^+^) on the right panel (MOI = 15, n = 3). E) As in D, flow cytometry plots (left) for WT and TMEM16F KO RMA cells ectopically expressing GFP (CT, control), R478A, or F518A TMEM16F mutants, after *Lm* infection for 2h, and the statistical analysis of PS exposure (Annexin V^+^PI^−^) and cell death (PI^+^) (right) (MOI = 15, n = 3). F) As in E, Annexin V and PI staining for the WT and TMEM16F KO RMA cells overexpressing GFP (CT) or scramblase domain chimeras (SCRD) ± *Lm* infection for 2 h (left). Quantification of PS exposure and cell death was on the right panel (MOI = 15, n = 6). All the experiments were performed for at least two times independently. Data are presented as mean ± SEM. The “n” represents the technical replicates. Statistical analysis was two‐way ANOVA for A to F. ns, not significant. ^*^ *p* < 0.05, ^**^ *p* < 0.01, ^***^ *p* < 0.001.

We have previously discovered the cellular protective role of TMEM16F from PM damage by LLO‐induced pore formation in vitro.^[^ ^30^ ^]^ However the underlying molecular mechanism during *Listeria* infection remains unknown. To study what kind of immune cells is under the protection of TMEM16F to resist cell lysis during *Listeria* infection, we co‐cultured the mouse splenocytes with *Lm*. Nearly all immune cells, including splenic macrophages, exhibited increased cell death after *Listeria* infection in the absence of TMEM16F, with the exception of CD8^+^ T cells (**Figure** **1**A). In addition, TMEM16F‐deficient bone marrow‐derived macrophages (BMDMs) and immortalized BMDM (iBMDM) displayed heightened sensitivity to *Listeria*‐induced cell death, as indicated by cell‐impermeable nucleic acid dye propidium iodide (PI) staining and lactate dehydrogenase (LDH) release (Figure 1B,C; Figure S1A,B, Supporting Information). These findings suggest that TMEM16F may have a potentially universal protective effect against *Listeria*‐induced cell death.

To further characterize the role of TMEM16F in counteracting *Listeria*‐induced cell death, we employed *Lm* to infect primary cells from mice and cell lines in vitro. Using Annexin V (which binds PS in the presence of Ca^2+^) and nucleic acid staining (PI or DAPI, to assess the PM integrity), we could distinguish the population of cells with intact plasma membranes (Annexin V^+^PI^−^) from the population with permeabilized PM (PI^+^). Of note, low concentration of DAPI used in this study could only stain cells with the permeabilized PM, same as PI (Figure S1C, Supporting Information).^[^ ^30^ ^]^ When co‐cultured with an increasing multiplicity of infection (MOI) of *Lm*, wild‐type (WT) thymocytes displayed more PS exposure, exhibiting substantial resistance to *Listeria*‐induced cell death, especially at low MOI of infection, whereas loss of TMEM16F led to significantly greater cell death (Figure S1D, Supporting Information). Additionally, thymocytes lacking TMEM16F displayed increased permeabilization, as evidenced by leakage of 5(6)‐Carboxyfluorescein diacetate N‐succinimidyl ester (CFSE) from preloaded cells and LDH release assay (Figure S1E,F, Supporting Information). Similar results were obtained in lymphoma cell line RMA as well. TMEM16F‐deficient RMA cells showed no PS exposure but increased PM permeabilization (Figure S1G–I, Supporting Information). The heightened cell permeabilization in TMEM16F KO cells was not due to their sensitivity to the bacterial infection because *Lm* infected both WT and TMEM16F KO cells identically, as indicated by the similar frequency of *Lm*‐GFP^+^ fractions (Figure S1J, Supporting Information).

Various cell death pathways have been described for cells infected by *Lm*.^[^ ^12^, ^13^, ^32^, ^33^ ^]^ Surprisingly, when using well‐established inhibitors of programmed cell death, including zVAD‐FMK (pan‐caspase inhibitor), Necrostatin‐2 (necroptosis inhibitor), YVAD‐FMK (caspase‐1 inhibitor), and Ferrostatin‐1 (ferroptosis inhibitor) during *Lm* infection, none were able to abolish or even reduce cell death in TMEM16F KO cells induced by *Lm* infection (Figure S1K, Supporting Information). This suggests that death in TMEM16F‐deficient cells induced by *Listeria* differs from known cell death pathways. To identify the virulent bacterial factors responsible for PS exposure and lethality from *Lm* infection, we infected thymocytes using *actA‐* (Δ*actA*, deletion of ActA) and *hly‐*null (Δ*hly*, deletion of LLO) *Lm* mutants, along with the control *Lm* strain. All these *Lm* strains infected WT and TMEM16F KO thymocytes equivalently (Figure S1L, Supporting Information). Control and *actA*‐deficient *Lm* elicited robust and similar levels of PS exposure in WT cells and cell death in TMEM16F‐deficient cells. In contrast, *hly*‐null *Lm* failed to do so in both WT and TMEM16F‐deficient cells (Figure S1M,N, Supporting Information).

Next, to uncover the molecular mechanisms of TMEM16F in counteracting *Lm*‐induced cell death, we first restored the TMEM16F expression in RMA TMEM16F‐deficient cells using a retrovirus‐based system (Figure S2A, Supporting Information). Re‐expression of TMEM16F enabled TMEM16F KO cells to externalize PS when treated with ionomycin (Figure S2B, Supporting Information). Concurrently, the restoration of TMEM16F expression significantly reinstated lipid scrambling ability in RMA TMEM16F KO cells upon *Lm* infection, leading to PS exposure instead of cell death (Figure 1D). These data support the role of TMEM16F in preserving PM integrity during *Lm* infection in a cell‐intrinsic manner.

TMEM16F has been reported to have multiple molecular functions, including Ca^2+^‐activated lipid scramblase and ion channels, particularly chloride and cation channels.^[^ ^35^, ^36^ ^]^ However, when using T16A(inh)‐A01, a previously reported chloride channel inhibitor for TMEM16F,^[^ ^46^ ^]^ we observed no alteration of PS exposure in WT cells nor cell death in TMEM16F KO cells (Figure S2C, Supporting Information). This rules out the involvement of the chloride channel function of TMEM16F in *Listeria* infection.

In addition to chloride channel activity, the phospholipid scramblase function was tested. We introduced one loss‐of‐function mutation (arginine 478 to alanine, R478A)^[^ ^47^ ^]^ and one gain‐of‐function mutation (phenylalanine 518 to alanine, F518A)^[^ ^48^ ^]^ of mouse TMEM16F into TMEM16F‐deficient RMA cells (Figure S2D, Supporting Information). Consistent with previous reports, flow cytometry of PS exposure induced by ionomycin confirmed R478A and F518A's function, with the R478A mutant failing to restore the lipid scrambling in TMEM16F‐null cells, whereas F518A could (Figure S2E, Supporting Information). Meanwhile, subjected to *Lm* infection, compared to empty GFP vector control, the F518A mutant but not the R478A mutant empowered TMEM16F‐null cells to scramble lipids and preserve the plasma membrane integrity (Figure 1E). To further validate that the lipid scrambling can preserve the plasma membrane integrity upon *Listeria* infection, mouse TMEM16A, the authentic TMEM16 family member with solely the chloride channel function, was fused with the previously identified scramblase domain (SCRD) from TMEM16F, forming SCRD chimeras.^[^ ^49^ ^]^ SCRD chimeras largely restored the lipid scrambling function triggered by ionomycin in TMEM16F KO RMA cells (Figure S2F, Supporting Information). Moreover, ectopic expression of SCRD chimeras in TMEM16F‐deficient RMA cells facilitated the PS externalization and saved cells from death upon *Listeria* infection (Figure 1F). Together, these results highlight that lipid scrambling by TMEM16F is capable of antagonizing the cell death induced by *Lm* infection, mostly due to LLO, thereby limiting the unconstrained release of intracellular content during membrane rupture.

### 2.2 The Cell‐Intrinsic Lipid Scrambling Mediate by TMEM16F Prevents the PM Leakage from *Lm*‐Elicited Injury

**Figure 2:**
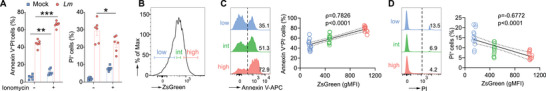
TMEM16F prevents the PM leakage from *Lm*‐elicited injury. A) Quantification of PS exposure (left) and cell death (right) of thymocytes treated with the low dose of ionomycin (1 µM) upon *Lm* infection (n = 6). B) Representative histogram of ZsGreen fluorescence in thymocytes isolated from TMEM16F‐ZsGreen reporter mice (fluorescent intensity arbitrarily defined as low, intermediate, and high). C, D) Representative histogram of Annexin V (C) and PI (D) staining in thymocytes population with low, intermediate, and high level of ZsGreen after *Lm* infection (left) and the correlation analysis between ZsGreen and Annexin V or PI (right) (n = 12 for C and D). All the experiments were repeated for at least twice independently. Data are presented as mean ± SEM. The “n” represents the technical replicates. Two‐way ANOVA for A, and Spearman correlation analysis for C and D. ^*^ *p* < 0.05, ^**^ *p* < 0.01, ^***^ *p* < 0.001.

Next, we aimed to investigate the potential connection between the lipid scrambling ability of TMEM16F and the maintenance of plasma membrane integrity. Using a low dose of ionomycin (1 µm), we induced mild lipid scrambling in WT thymocytes without significant cell death. Under these conditions, cells exhibited more pronounced PS exposure and, simultaneously, increased resistance to *Lm*‐induced cell death (**Figure** **2**A). Additionally, due to the lack of availability of anti‐TMEM16F antibody for immunofluorescent staining, we generated a *Tmem16f‐ZsGreen* reporter mouse by inserting *ZsGreen* sequence into the last exon of *Tmem16f* (Figure S3A, Supporting Information). Thymic ZsGreen expression detected by flow cytometry reflected the endogenous expression of TMEM16F in this mouse (Figure S3B, Supporting Information). We arbitrarily defined ZsGreen high, intermediate, and low expression populations in thymocytes, representing the level of TMEM16F from high to low (Figure 2B). Analyzing their responses to *Lm* infection, we found TMEM16F expression level positively correlated with PS exposure ability (Figure 2C). As a result, ZsGreen^hi^ cells were more resistant to *Lm*‐induced death (Figure 2D). Collectively, these results support the idea that the lipid scrambling prevents PM leakage from *Lm*‐induced damage, representing a cell‐intrinsic lipid scrambling mechanism.

### 2.3 Extensive Plasma Membrane Lipids Re‐Shuffling by TMEM16F During *Listeria* Infection

**Figure 3:**
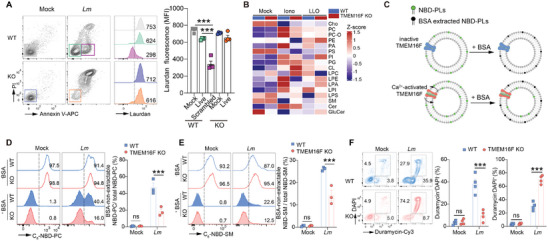
Extensive plasma membrane lipids reshuffling by TMEM16F in response to *Listeria* infection. A) Representative flow cytometry plots of Annexin V and PI (left) in WT and TMEM16F KO RMA cells after *Lm* infection and the histogram of Laurdan dye fluorescence (middle panel, numbers depict the MFI) from the indicated gates. Quantification of Laurdan dye fluorescence is on the right panel (n = 4) (Mock, uninfected; Live, Annexin V^−^PI^−^; Scrambled, Annexin V^+^PI^−^). B) The heatmap of lipidomic analysis of plasma membrane purified from WT and TMEM16F KO thymocytes treated with or without ionomycin or LLO (n = 3‐4). C) Schematic diagram of TMEM16F‐mediated membrane lipid scrambling detected by NBD‐labeled lipid probe after *Lm* infection. D,E) As illustrated in (C), representative histogram (left) and percentage of scrambling (right) for NBD‐PC (D) or NBD‐SM (E) in WT and TMEM16F KO RMA cells after *Lm* infection. Percent of NBD^+^ population was indicated on the histogram. n = 3. F) As in (A), representative flow cytometry plots of duramycin and DAPI staining (left) for WT and TMEM16F KO RMA cells after *Lm* infection, with the statistical analysis on the right (n = 4). All the experiments were repeated for at least two times independently and data are presented as mean ± SEM. The “n” represents the technical replicates. Unpaired Student's *t* test for A and two‐way ANOVA for D to F. ns, not significant. ^***^ *p* < 0.001.

TMEM16F‐mediated lipid scrambling happens during *Listeria* infection. To delve deeper into the specifics of how TMEM16F modulates plasma membrane lipids in response to intracellular bacterial infection‐induced PM damage, we initiated our investigation using Laurdan dye, a fluorescent probe that reflects plasma membrane lipid order.^[^ ^50^, ^51^ ^]^ Intriguingly, we observed a pronounced decrease of Laurdan signal in living scrambled cells (Annexin V^+^ PI^−^) during *Listeria* infection, a phenomenon not witnessed in TMEM16F‐deficient cells (**Figure** **3**A). Furthermore, scrambled cells displayed a notable increase in merocyanine 540 (MC540) fluorescence, a marker for evaluating membrane lipid gel/liquid status (Figure S4A, Supporting Information).^[^ ^52^ ^]^ These results from lipophilic probes suggested the substantial modifications of plasma membrane lipids.

To gain a comprehensive understanding of the specific lipids modulated by TMEM16F‐dependent lipid scrambling, we conducted lipidomics through mass spectrometry (MS) on plasma membrane samples purified from thymocytes treated with ionomycin and LLO. Unstimulated samples from WT and TMEM16F KO thymocytes exhibited highly similar lipid profiles, indicating minimal effects of TMEM16F on PM lipids before activation. While ionomycin and LLO treatment induced substantial alterations in lipid composition on the plasma membrane, possibly due to endocytosis and exocytosis, cells lacking TMEM16F‐mediated lipid scrambling displayed fewer modifications compared to WT cells, particularly in membrane lipids such as phospholipids, sphingolipids, and sterols (Figure 3B; Figure S4B–G, Supporting Information). Given that TMEM16F is a bona fide phospholipid scramblase, we sought to understand that lipids are directly affected by TMEM16F during *Listeria* infection. Beyond phosphatidylserine (PS), phosphatidylcholine (PC), sphingomyelin (SM), and phosphatidylethanolamine (PE) were scrambled by TMEM16F between the inner and outer layers of PM when cells were infected with *Lm*, as shown by NBD‐PC, NBD‐SM, and duramycin staining, respectively (Figure 3C–F). These findings underscore the extensive reshuffling of plasma membrane lipids by TMEM16F during *Listeria* infection, contributing to the preservation of PM integrity by altering PM fluidity.

### 2.4 Pro‐Inflammatory Response and Aberrant Lipid Cumulation Elicited by *L. Monocytogenes* in the Loss of TMEM16F

**Figure 4:**
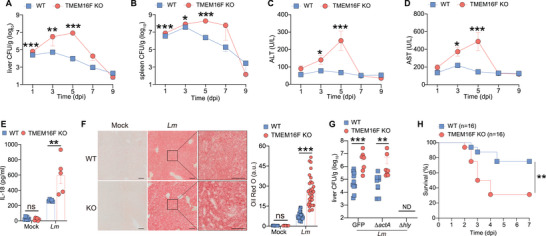
Pro‐inflammatory response and tissue damage elicited by *L. monocytogenes* infection in the loss of TMEM16F. A,B) Bacterial load (CFU) in the liver and spleen from WT and TMEM16F KO mice at indicated time post infection. Pooled data from three independent experiments, n = 8‐16. C,D) Serum ALT and AST from mice as in (A and B). Pooled data from two independent experiments, n = 6‐10. E) Level of IL‐18 in serum obtained from uninfected (Mock) and *Lm*‐infected WT and TMEM16F KO mice at 3 dpi (n = 5‐6). F) Oil Red O staining of liver sections from uninfected (Mock) and *Lm*‐infected WT and TMEM16F KO mice at 3 dpi (left) and quantification in (F) (a.u., arbitrary unit) (right). Mock, n = 10; *Lm*, n = 30. Scale bar, left 100 µm, right 50 µm. G) Bacterial burden in the liver after *Lm* infection at 3 dpi. ND, not detected. Δ*actA*, *actA* KO; Δ*hly*, LLO KO; GFP, control *Lm*. n = 7‐11. H) Survival curve of WT and TMEM16F KO mice after infection by *actA* KO *Lm*. Data were pooled from three independent experiments (n = 16). All the experiments were performed for at least two independent times. Data are presented as mean ± SEM. Statistical analysis by two‐way ANOVA with Dunnett's multiple comparisons test (E,F), logrank (Mantel–Cox) test (H) or two‐tailed Mann–Whitney test (A–D and G). ns, not significant. ^*^ *p*<0.05, ^**^ *p*<0.01, ^***^ *p*<0.001.

Germline knockout (KO) of TMEM16F mice behave more susceptible to *Lm* infection than WT mice.^[^ ^30^ ^]^ However the consequences of TMEM16F‐deficiency in response to *Lm* infection in vivo have not been characterized. To address it, we initiated our investigation by injecting a sublethal dose (10^4^ CFU/mouse) of *Lm* strain 10403s, obtained from log‐phage culture, into TMEM16F germline KO mice and their WT littermates controls intravenously (iv). The bacterial number was identical in the liver 20 min after injection, indicating equal susceptibility to *Listeria* infection in both mouse groups (Figure S5A, Supporting Information). Subsequent evaluation of bacterial burden in the liver and spleen from 1 to 9 days post‐infection (dpi), the principal organs infected via the intravenous route, revealed that TMEM16F‐deficient mice exhibited a significantly higher bacterial burden compared to their WT counterparts (**Figure** **4**A,B). Notably, while the bacterial load decreased after 3 dpi in WT mice, it continued to rise until 5 dpi in TMEM16F KO mice (Figure 4A,B). Furthermore, utilizing GFP‐expressing *Lm* to track cells infected by bacteria in vivo, we identified that the immune cells infected by *Listeria* six hours after infection in various organs are predominantly myeloid cells, including macrophages, neutrophils, and monocytes, as well as the B cells in the liver (Figure S5B–E, Supporting Information). Importantly, cells infected by *Listeria* were equivalent in WT and TMEM16F KO mice, suggesting comparable bacterial entry in both cell types in vivo (Figure S5B–E, Supporting Information). Taken together, this kinetic analysis of bacterial burden indicates that TMEM16F plays a role in controlling *Listeria*, particularly in the early phase of infection.

As a result, TMEM16F KO mice experienced significant liver damage and an intensified pro‐inflammatory response following *Listeria* infection, as evidenced by heightened alanine transaminase (ALT) and aspartate transaminase (AST) activities, along with increased levels of the inflammatory cytokine IL‐18 in the serum (Figure 4C–E). Additionally, staining of Oil Red O in fresh liver samples demonstrated a substantial accumulation of lipids induced by *Listeria* infection, a phenomenon significantly aggravated in the absence of TMEM16F (Figure 4F). This suggests an abnormal hepatocyte metabolism in mice lacking TMEM16F upon *Lm* infection.

TMEM16F is exclusively located on the PM,^[^ ^41^, ^45^ ^]^ and *Listeria* injure the PM mainly by secreting the pore‐forming toxin LLO and transmitting between the cells via ActA.^[^ ^28^ ^]^ To discern which *Listeria* virulence factor is involved in the protective effect of TMEM16F in vivo, we employed *Lm* strains with genetic deletion of ActA (Δ*actA*) or LLO (Δ*hly*) to infect the mice.^[^ ^53^ ^]^ Consistent with previous reports,^[^ ^9^ ^]^ loss of ActA did not compromise the *Lm* infection in the liver of WT mice. Similar to control *Lm* strain, TMEM16F‐deficient mice retained much higher liver bacteria load than WT mice during Δ*actA Lm* infection. In contrast, LLO‐deficient *Listeria* was rapidly cleared at 3 dpi in both genotypes (Figure 4G), ruling out a role of ActA‐mediated cell‐cell spreading but suggesting potential involvement of LLO‐induced PM lesions in the severe pathological consequences observed in TMEM16F KO mice after infection. Given the life‐threatening nature of *Lm*, we investigated if TMEM16F could protect mice from *Listeria*‐induced mortality by conducting a survival experiment with a lethal dose of ActA‐deficient *Lm* (10^6^ CFU/mouse). TMEM16F KO mice succumbed to infection more rapidly and at a higher mortality rate than their control littermates (Figure 4H). Thus, TMEM16F protects the mice from higher bacterial load and death after *Lm* infection, possibly by regulating inflammatory response and lipid metabolism in the liver.

### 2.5 The Absence of TMEM16F Triggers an Exacerbated Pro‐Inflammatory Response and Disrupted Lipid Metabolism in the Liver in Response to *Lm* Infection

**Figure 5:**
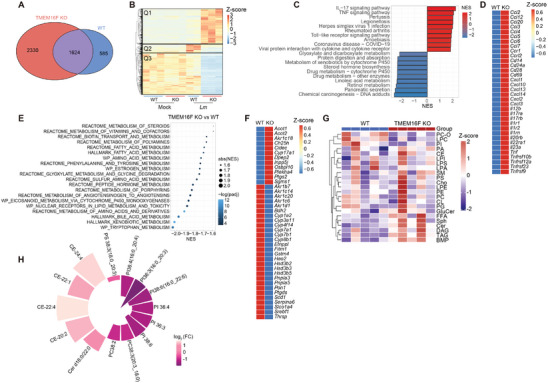
Loss of TMEM16F results in excessive pro‐inflammatory response and dysregulated lipid metabolism in the liver in response to *Lm* infection. All samples used in ‐omics analysis were acquired at 3 dpi. A) Venn diagram showed differentially expressed genes (DEGs) in WT and TMEM16F KO mice after *Lm* infection. B) Heatmap of gene transcriptional profiles obtained from the liver of uninfected (Mock) and *Lm*‐infected WT and TMEM16F KO mice. C) Top enriched KEGG pathways of DEGs in (A). D) Heatmap of pro‐inflammatory genes in *Lm*‐infected WT and TMEM16F KO mice. E) The metabolic pathway enrichment analysis between WT and TMEM16F KO mice based on the Gene Signature Enrichment Analysis (GSEA) database. F) Heatmap of genes involved in lipid metabolism from the liver of *Lm*‐infected WT and TMEM16F KO mice. G) Liver lipidomic analysis in *Lm*‐infected WT and TMEM16F KO mice (n = 5). Heatmap analysis of lipids between WT and TMEM16F KO samples. H) Individual lipids with p_adj_<0.05 between WT and KO groups (n = 5).

To further characterize the hepatic alteration after *Lm* infection, we conducted RNA sequencing on liver samples from WT and TMEM16F KO mice at 3 dpi. Principal component analysis (PCA) illustrated nearly identical transcriptome in uninfected WT and TMEM16F liver, which diverged significantly after infection (Figure S6A, Supporting Information). TMEM16F KO mice exhibited a considerably higher number of differentially expressed genes (DEGs) compared to WT counterparts after infection (**Figure** **5**A). Heatmap analysis of gene transcription profiles confirmed minimal differences at transcriptomic level between uninfected WT and TMEM16F liver. However, after *Lm* infection, TMEM16F KO mice displayed a profound modulation in gene expression profiles, albeit to a lesser extent in the WT control group (Figure 5B). Further categorization of DEGs between WT and TMEM16F KO mice after infection revealed three distinct categories (Q1‐Q3). Gene ontology (GO) analysis indicated that the majority of upregulated genes in the absence of TMEM16F were associated with immune responses, while downregulated genes were implicated in cell metabolism (Figure 5C; Figure S6B, Supporting Information). Upregulation of pro‐inflammatory genes in TMEM16F KO mice pointed to heightened inflammation, aligning with our previously reported results (Figure 5D). Subsequent GO biological process gene enrichment analysis on these three gene categories separately revealed cytokine production and cell migration enrichment in infected TMEM16F KO mice in Q1, enhanced antigen presentation and cell‐cell adhesion in infected WT mice in Q2, and downregulated metabolic and biosynthetic processes in infected KO mice in Q3 (Figure S6C–E, Supporting Information). These findings suggest that TMEM16F deficiency intensifies the pro‐inflammatory response, subsequently leading to aberrant cellular metabolism, particularly in lipid metabolism, and affecting further adaptive immunity.

TMEM16 family contains ten family members. TMEM16A and 16B are chloride channels without any scramblase function. TMEM16E, 16H, and 16K are absent on the plasma membrane. TMEM16C, 16D, 16F, 16G and 16J are Ca^2+^‐dependent scramblases.^[^ ^54^ ^]^ In our bulk RNAseq results from WT and TMEM16F‐deficient mice, we only observed highly expressed plasma membrane scramblase TMEM16F in the liver (Figure S6F, Supporting Information). Though we are not able to exclude other TMEM16 family member's involvement in liver damage induced by *Lm* infection, TMEM16F exhibits protective role against *Lm*‐elicited liver damage.

Additionally, gene set enrichment analysis (GSEA) unveiled enriched pathways in various metabolic processes, including lipids, amino acids, and hormones, indicating systemic alterations in cell metabolism in the injured liver in the absence of TMEM16F following infection (Figure 5E). Notably, genes associated with lipid metabolism were predominantly downregulated in TMEM16F KO mice (Figure 5F). This aligns with previous reports suggesting that bacterial infection‐induced inflammation can result in dysregulated metabolism.^[^ ^55^, ^56^ ^]^ As observed in the WT group, *Lm* infection led to fatty liver, and TMEM16F deficiency exacerbated lipid accumulation (Figure 4F). This suggests that dysregulated metabolism after *Lm* infection is primarily attributed to the heightened inflammation in mice lacking TMEM16F.

Furthermore, we performed lipidomics to scrutinize the lipid profiles post *Lm* infection. Despite considerable variations among individual mice, we observed increased diglycerides (DAG) and triglycerides (TAG), along with alterations in specific phospholipids (PLs) and cholesteryl esters (CE) in the liver of TMEM16F KO mice (Figure 5G,H; Figure S6G, Supporting Information). Thus, our transcriptomic results support that the elevated bacterial titers in TMEM16F KO mice post *Lm* infection elicited an excessive inflammatory response, potentially leading to dysregulated hepatic metabolism and exacerbated liver injury.

### 2.6 TMEM16F Expressed in Kupffer Cells is key to Protect from Damage upon *Lm* Infection

*Listeria* has the capacity to systematically infect various organs throughout the body, including the ability to breach the blood‐brain and placental barriers.^[^ ^4^, ^8^, ^9^, ^57^, ^58^ ^]^ To discern the specific cell types in which TMEM16F acts against *Listeria* infection, we initially established bone marrow (BM) chimeric mice by transplanting BM cells from both WT and TMEM16F KO donors into sub‐lethally irradiated WT and TMEM16F KO recipients, respectively. Thirty‐five days later, these BM chimeric mice were challenged by *Lm*, and subsequent examination revealed significantly higher CFU in the livers of irradiated WT recipients receiving TMEM16F‐deficient BM cells, akin to TMEM16F KO mice receiving TMEM16F‐deficient BM cells, in comparison to the control group (WT BM cells transferred into WT recipients) (Figure S7A, Supporting Information). This suggests a crucial contribution of TMEM16F in hematopoietic cells to resistance against *Listeria* infection.

**Figure 6:**
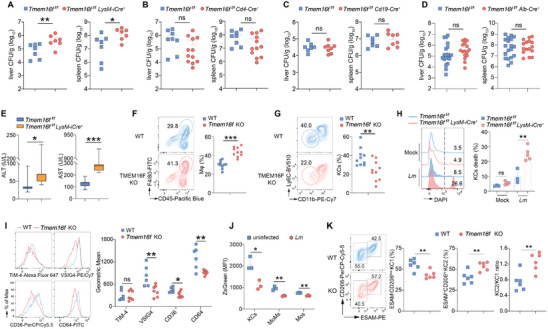
Depletion of Kupffer cells upon *Lm* infection due to the ablation of TMEM16F. (A to D) Quantification of bacterial load in the liver and spleen from various tissue‐specific *Tmem16f* KO mice and their corresponding littermate controls at 3 dpi. Data were pooled from at least two independent experiments. n = 7 for (A), n = 8‐12 for (B), n = 7–8 for (C), n = 16‐20 for (D). E) Quantification of serum ALT and AST activity from myeloid‐specific *Tmem16f* KO mice at 3 dpi. Data were pooled from at least two independent experiments. n = 7. F) Flow cytometry analysis of macrophages (CD45^+^F4/80^+^) in the liver from *Lm‐*infected WT and TMEM16F KO mice at 3 dpi. Data were pooled from two independent experiments (n = 10). Mϕ, macrophages. G) Flow cytometry analysis of cells gated on (F). KCs are gated on CD11b^lo^Ly6C^lo^. n = 10. H) Representative histogram (left) and percentage of KCs death (right) after *Listeria* infection for 1 h in vitro. Cells were isolated from *LysM‐iCre‐Tmem16f^fl/fl^* and *Tmem16f^fl/fl^* control mice. MOI = 10, n = 3–5. I) Expression of typical KCs (CD45^+^F4/80^+^CD11b^+^CLEC2^+^) surface markers (n = 6–7) in WT and TMEM16F KO mice after *Lm* infection at 3 dpi. J) TMEM16F expression, represented by ZsGreen fluorescence, in uninfected and *Lm*‐infected *Tmem16f‐ZsGreen* reporter mice (n = 3). MoMs, monocyte‐derived macrophages (CD45^+^F4/80^+^CD11b^hi^Ly6C^int^); Mos, inflammatory monocytes (CD45^+^F4/80^+^CD11b^int^Ly6C^hi^). K) Flow cytometry analysis of KCs subsets in the liver from *Lm‐*infected WT and TMEM16F KO mice at 1 dpi. Data were pooled from two independent experiments (n = 6). KC1 are gated on CD45^+^CD11b^+^F4/80^+^CD64^+^TIM‐4^hi^CD206^low^ESAM^−^. KC2 are gated on CD45^+^CD11b^+^F4/80^+^CD64^+^TIM‐4^hi^CD206^+^ESAM^+^. All the experiments were repeated at least twice. Data are presented as mean ± SEM. Representative flow cytometry results were shown on the left panel and statistical analysis were on the right panel (F to I, K). Each dot represents one mouse. Two‐tailed Mann–Whitney test (A to E), unpaired Student's *t* test (F, G, I–K) or two‐way ANOVA (H) were used for statistical analysis. ns, not significant. ^*^ *p*< 0.05, ^**^ *p*< 0.01, and ^***^ *p*<0.001.

To further identify the immune cell types responsible for TMEM16F‐mediated protection, we generated conditional TMEM16F KO mice by crossing *Tmem16f* floxed mice (*Tmem16f ^f/f^*) with various *Cre* recombinase expressing mice (*LysM‐iCre* to have myeloid‐specific *Tmem16f* deletion, *Cd4‐Cre* to delete *Tmem16f* in T cells, and *Cd19‐Cre* to deplete *Tmem16f* in B cells) (Figure S7B–D, Supporting Information). Upon *Listeria* infection at day 3, increased bacterial loads were observed in LysM‐iCre‐*Tmem16f ^f/f^* mice but not in CD4‐Cre *Tmem16f ^f/f^* or CD19‐Cre *Tmem16f ^f/f^* mice, compared to their respective WT littermates (**Figure** **6**A–C). Moreover, given that hepatocytes are the majority in the liver whether TMEM16F in hepatocytes playing the protective effect remains unknown. Upon *Listeria* infection at day 3, we found equal liver bacterial load and same level of serum AST and ALT in both Alb‐Cre *Tmem16f ^f/f^* and their WT littermates, suggesting that TMEM16F in hepatocytes may not be involved in the control of *Lm* infection (Figure 6D; Figure S7E, Supporting Information). All the conditional TMEM16F KO mice results indicate that TMEM16F in myeloid immune cells plays a role in protecting against *Lm* infection at early stages, while no such involvement is noted in T and B cells or hepatocytes (Figure 6A–D). Simultaneously, similar to our previous observations in germline KO mice, the loss of TMEM16F in myeloid cells led to exacerbated liver injury after infection, as evidenced by higher serum levels of AST and ALT, an increased level of the IL‐18 in the serum, and a substantial accumulation of lipids by staining of Oil Red O in liver samples (Figure 6E; Figure S7F–G, Supporting Information). These findings underscore the specific contribution of TMEM16F in myeloid cells to protection against bacteria‐induced liver damage.

Myeloid cells, including macrophages and neutrophils, play crucial roles in the early control of *Lm* infection. Consistent with previous studies,^[^ ^59^ ^]^ our results found ≈95% of the bacteria injected intravenously were trapped in the liver within 10 min (Figure S7H, Supporting Information). Liver resident macrophages, known as Kupffer cells (KCs), were identified as the primary host cells in the early phase of *Lm* infection.^[^ ^60^, ^61^, ^62^ ^]^ Our hypothesis centered on the significant contributions of TMEM16F in controlling *Listeria* infection through myeloid cells. To validate this hypothesis, we conducted the analysis of myeloid cells in the liver. At 3 dpi, germline and LysM‐iCre TMEM16F KO mice exhibited higher counts of total macrophages (CD45^+^F4/80^+^), inflammatory monocytes (CD45^+^CD11b^int^F4/80^lo^Ly6C^hi^), and neutrophils (CD45^+^CD11b^+^Ly6G^+^) compared to WT controls (Figure 6F; Figure S7I–L, Supporting Information). The increased recruitment of monocytes and neutrophils is likely a consequence of more severe inflammation and liver injury in the absence of TMEM16F. Similarly, we observed fewer Gr‐1^+^ monocytes and Ly6G^+^ neutrophils in the bone marrow of TMEM16F KO mice after *Lm* infection, suggesting elevated egress of these cells from the BM in these mice (Figure S7M–N, Supporting Information). To investigate whether monocytes and neutrophils migrated to damaged foci during infection, we adoptively transferred BM cells from *Cx3cr1‐Cre* ^+^ tdTomato reporter mice into WT and TMEM16F KO mice at 3 dpi. More *Cx3cr1*‐tdTomato^+^ myeloid cells were recovered from the spleens in TMEM16F KO mice compared to WT mice, providing support for an increased infiltration of myeloid cells into the damaged tissues in TMEM16F KO mice (Figure S7O, Supporting Information).

As a facultative intracellular bacterium, *L. monocytogenes* primarily infects Kupffer cells (KCs), during the early stage of infection.^[^ ^12^ ^]^ In *Listeria* infection, KCs undergo cell death and subsequently re‐populate from monocytes recruited into the liver.^[^ ^12^ ^]^ Surprisingly, we found a more pronounced depletion of KCs (F4/80^+^Ly6C^lo^CD11b^lo^) in the absence of TMEM16F compared to their control littermates at 3 dpi, at both percentage and absolute number (Figure 6G; Figure S8A,B, Supporting Information). Notably, this was not attributed to a proliferation defect of TMEM16F‐deficient KCs, as indicated by Ki‐67 staining (Figure S8C, Supporting Information), suggesting that the increased loss of KCs in TMEM16F KO mice might be owing to cell death. To confirm it, we found that TMEM16F‐deficient KCs were more susceptible to *Lm*‐infection induced cell death, compared to control KCs (Figure 6H). In line with it, KCs with higher TMEM16F expression exhibited more resistant to *Lm*‐induced death (Figure S8D, Supporting Information).

Additionally, KCs in TMEM16F KO mice exhibited an altered expression pattern for typical surface markers, including VSIG4, CD36, and CD64, implying potential modulation of KCs functions, possibly influenced by excessive inflammation and dysregulated metabolism (Figure 6I). Furthermore, using our *Tmem16f‐ZsGreen* reporter mouse, we observed a decrease in endogenous TMEM16F expression in hepatic myeloid cells, particularly in KCs after *Lm* infection (Figure 6J; Figure S8E, Supporting Information). The reduced TMEM16F expression in these cells may exacerbate tissue injury upon infection. Moreover, further analysis revealed that KC subsets were different between WT and TMEM16F upon *Lm* infection, suggesting that KC subsets may respond to *Lm* infection distinctively (Figure 6K). Overall, the lower quantity of KCs with altered marker expression in mice lacking TMEM16F may compromise the antibacterial ability, consequently leading to the elevated bacterial burden and tissue damage mentioned earlier.

### 2.7 The Absence of TMEM16F In Vivo Results in KCs Experiencing PM Rupture and Fragmentation During *Lm* Infection

**Figure 7:**
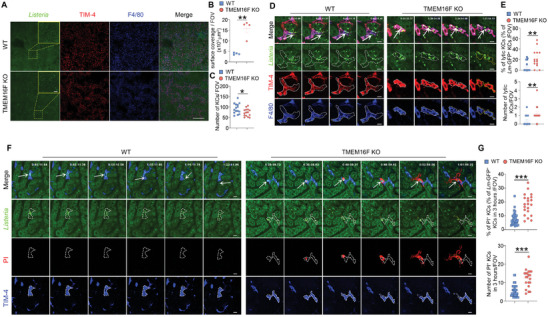
KCs PM rupture and fragmentation during *Lm* infection due to the loss of TMEM16F in vivo. A) Intravital microscopy images (left) of the liver infected with the GFP‐expressing *Lm* (5×10^7^ bacteria) at 12 h post‐infection. Scale bar, 500 µm in tile scan and 50 µm in zoom. B) Quantification of the surface coverage of GFP‐expressing *Lm* in WT and TMEM16F KO mice shown in (A) (n = 4). C) Quantification of the KCs number per field of view (FOV) after GFP‐expressing *Lm* infection. Mice were infected with 5×10^7^ CFU GFP^+^ *Lm* intravenously, and images were taken 12 h post‐infection. n = 15. D) Intravital microscopy images of Kupffer cell lysis (F4/80^+^TIM‐4^+^) after GFP‐expressing *Lm* infection in vivo. Mice were infected with 5×10^7^ CFU GFP^+^ *Lm* intravenously, and images were taken 12 h post‐infection. Bright green dots were GFP^+^ *Listeria* and white arrows indicated the *Lm*‐containing KCs. The white lines represent the cell membrane boundaries in WT and KO KCs, and the yellow lines represent cell fragmentation in KO KCs. Scale bar, 10 µm. E) Quantification of the lytic KCs in WT and TMEM16F KO mice shown in (D) (n = 14‐17). F) Detection of KCs (TIM‐4^+^) plasma membrane rupture elicited by GFP^+^ *Lm* infection in vivo. Mice were infected with 1×10^8^ CFU GFP^+^ *Lm* intravenously, and nucleic acid staining dye PI was injected 1 h post‐infection. Images were taken right after PI injection. Plasma membrane rupture was monitored by red fluorescence of PI, when the plasma membrane integrity was lost. White arrows represent the location where the bacteria escape from the KCs. The white lines represent the PM boundaries in WT and KO KCs, while the yellow lines represent PM components derived from cell rupture in KO KCs. Scale bar, 10 µm. G) Quantification of the ruptured KCs in WT and TMEM16F KO mice shown in (F) (n = 21‐46). Data are from at least two independent experiments and presented as mean ± SEM. Two‐tailed Mann–Whitney test for panels B, C, E and G. ^*^ *p*<0.05, ^**^ *p*<0.01, ^***^ *p*<0.001. See related Supplemental Videos S1 and S2 (Supporting Information).

To further elucidate the role of TMEM16F in KCs during bacterial infection, we employed intravital microscopy to visualize the dynamics of *Listeria* infection in vivo. GFP‐labeled *Lm*, administered intravenously, was rapidly and almost exclusively captured by KCs in the liver, peaking at 5 min post‐infection (pi), in alignment with previous reports (Figure S9A,B, Supporting Information).^[^ ^12^ ^]^ KCs in both WT and TMEM16F KO mice demonstrated equal efficiency in capturing *Lm* upon their entry into the circulation (Figure S9B, Supporting Information). However, at 12 h pi, TMEM16F KO mice exhibited a more loading of bacteria into the liver compared to WT mice (**Figure** **7**A,B). This was concomitant with a significantly reduced number of KCs in the liver of TMEM16F KO mice, suggesting that TMEM16F may play a crucial role in preventing KC death and thereby limiting *Lm* evasion from these phagocytes (Figure 7A,C). Subsequently, we performed real‐time imaging of mouse liver to monitor the dynamic interactions between KCs and *Lm* at this time point. While occasional escape of *Lm* from KCs was observed in WT mice, this process did not lead to obvious membrane rupture of KCs. In stark contrast, *Lm*‐infected KCs in TMEM16F KO mice frequently exhibited robust membrane rupture and fragmentation, resulting in the rapid spread of intracellular bacteria (Figure 7D,E; Video S1, Supporting Information).

To further validate these observations, we assessed the plasma membrane integrity in *Lm*‐infected KCs in real‐time by intravenously injecting the cell‐impermeable nucleic acid dye propidium iodide (PI) one hour after *Lm*‐GFP infection. Within the initial two hours after PI injection, some TMEM16F‐deficient KCs, but not WT KCs exhibited PI staining by suddenly emitting red fluorescence. The PI staining pattern initially concentrated inside the cells (likely the nuclei) and subsequently spread throughout the cells, extending into the outer space of the KCs (Figure 7F,G; Video S2, Supporting Information). This pattern suggested plasma membrane breach followed by a process of cell lysis in *Lm*‐infected TMEM16F deficient KCs. As a consequence, there were significantly more infectious foci in the liver of TMEM16F KO mice compared to WT mice at 48 h post infection, consistent with the observations of increased tissue injury and bacterial burden in the absence of TMEM16F (Figure S9C,D, Supporting Information). These findings provide a detailed understanding of the role of TMEM16F in the bacteria–host interaction in vivo. In the absence of TMEM16F, KCs became more fragile, exhibiting PM rupture and fragmentation after *Lm* infection, resulting not only the loss of KCs in numbers, but also in the substantial release of danger signal molecules from dead corps. This unconstrained cell lysis released intracellular contents, ultimately fueling pro‐inflammatory responses in the liver.

## 3 Discussion

Food‐borne *Lm* continues to pose a significant challenge to public health since its identification.^[^ ^63^ ^]^ Despite extensive studies on *Lm* as a model bacterium, our understanding of its biology and pathology remains limited, particularly regarding the interaction of bacterium and host at the cellular and systemic level.^[^ ^58^, ^63^, ^64^, ^65^ ^]^ Through the use of mouse genetics and cell biological methods, we have uncovered an overlooked cell‐autonomous defense mechanism crucial in the acute phase of listeriosis. The controlled release of pro‐inflammatory signals through TMEM16F‐mediated lipid scrambling proved to be protective, shielding mice from excessive inflammation, dysregulated metabolism, and immunopathological outcomes triggered by *Lm* infection. Additionally, KCs relied on TMEM16F to protect themselves from lytic rupture in the liver caused by infection. Mechanistically, the extensive lipid scrambling by TMEM16F altered the PM fluidity, ensuring the PM integrity. Thus, lipid scrambling emerges as a key cell‐intrinsic player in harnessing inflammation and metabolism during infection to optimize the host's anti‐bacterial immune responses.

Liver‐resident macrophages undergoing necroptosis upon *Lm* infection play a crucial role in balancing anti‐bacterial inflammation and tissue homeostasis.^[^ ^12^ ^]^ Beyond that, our study delves into the intricate interplay between intracellular *Lm* and host cells on the plasma membrane in vivo. The absence of TMEM16F significantly heightened susceptibility to *Lm* infection in mice, resulting in exacerbated tissue damage, heightened inflammation, and perturbed liver metabolism. Consistent with previous findings, we found pronounced lytic cell death when cells were unable to execute lipid scrambling on PM during *Lm* infection. Intravital confocal microscopy revealed a significantly increased incidence of plasma membrane rupture and fragmentation in KCs of TMEM16F knockout mice compared to WT controls. This underscores the crucial role of host cell TMEM16F in countering the injury by *Lm*, thereby restraining the intracellular danger signaling molecules unleashed, and ensuring the cell survival. The pathological consequences were more severe in the absence of TMEM16F.

We also noted that the TMEM16F expression decreased upon *Lm* infection (Figure 6J), suggesting a potential strategy by bacteria to exploit lower TMEM16F levels for enhanced infection in the host. Controlled leakage of intracellular contents serves as a sufficient damage associated molecular patterns (DAMP) signal in WT mice, recruiting monocytes and neutrophils to inflammatory foci, clearing the bacteria, and re‐establishing tissue homeostasis. Conversely, the unbridled release of intracellular components triggers unrestrained inflammatory response to bacterial infection and disrupts hepatic metabolism in the absence of TMEM16F. Consequently, TMEM16F KO hosts suffered more severe immunopathological consequences, potentially leading to death. Notably, the primary protective role of lipid scrambling by TMEM16F appears to stem from immune cells, suggesting that immune cells may heavily depend on TMEM16F to mount an effective defense against *Listeria* attacks.

In the context of PM damage, our prior research has already illuminated the role of TMEM16F in safeguarding PM integrity in response to Ca^2+^ influx triggered by pore formation in vitro.^[^ ^30^ ^]^ While it was evident that TMEM16F‐deficient cells succumb to various PM injuries, including those induced by LLO, the in vivo role of TMEM16F during Listeria infection and the underlying molecular mechanisms have remained elusive. This study provides a comprehensive exploration of these molecular mechanisms and sheds light on the immunopathological implications of TMEM16F in bacterial infection. First, we found that TMEM16F exerts its protective effect on PM integrity primarily through its phospholipid scramblase function, independent of its anion channel properties. Furthermore, the SCRD chimeras were capable of facilitating the lipid scrambling and conferring the protective role to some extent, even though the ion channel conductance and lipid scrambling of TMEM16F seem to be unassociated.^[^ ^49^, ^66^ ^]^ Arginine 478 site is critical for lipid scrambling activity of TMEM16F, particularly for PS exposure. The R478A mutant of TMEM16F displays compromised PS exposure and reduced production of giant plasma membrane vesicles (GPMVs), without affecting Ca^2+^ influx.^[^ ^47^, ^67^ ^]^ Notably, the R478A mutant retains the capability to internalize NBD‐PC and NBD‐PS, indicating only partial impairment of its lipid scrambling ability. In contrast, the F518 site of TMEM16F functions as an inner activation gate. Mutation of phenylalanine to lysie at F518 can spontaneously activate TMEM16F scrambling, as evidenced by PS exposure using Annexin V staining.^[^ ^48^, ^68^ ^]^ Our results with these loss‐ and gain‐function of mutations establish the correlation between lipid scrambling and PM integrity maintenance. Thirdly, lipid scrambling by TMEM16F not only counteracts the PM permeabilization during infection, but more importantly, prevents the sudden release of the intracellular DAMP molecules, which are the pro‐inflammatory drivers. Recent advancements suggest that structural changes during lipid scrambling are accompanied by the opening of a pore that promotes the ion conductance in the same protein conformation.^[^ ^69^ ^]^ Therefore, it is plausible that direct Ca^2+^ influx through TMEM16F might synergize with Ca^2+^ from damaged sites, enhancing the local activation of lipid scrambling. These results might allow us to conclude that the lipid scrambling by TMEM16F represents a novel mechanism in maintaining plasma membrane integrity upon intracellular bacterial infection. However, the exact mechanism by which lipid scrambling maintains plasma membrane integrity upon damage remains an open question.

Several ways of cell death for host cells have been reported during *Lm* infection, including cell‐intrinsic programmed cell death necroptosis, pyroptosis, apoptosis, and cell‐extrinsic death.^[^ ^9^, ^12^, ^13^, ^33^ ^]^ However, in our in vitro experimental conditions, we observed that cell death in the absence of TMEM16F primarily resulted from the membrane damage caused by LLO, and a short period of time infection with *Listeria* has not initiated significant death in WT cells yet. This suggests that, in addition to the intricate programmed cell death pathways triggered by *Lm* infection, the maintenance of plasma membrane integrity, dependent on lipid scrambling mediated by TMEM16F, is crucial for cell survival when facing direct LLO injury. Compared to other innate immune recognition mechanisms, such as Toll‐like receptors via pattern recognitions and cGAS‐STING by sensing cytosolic DNA, plasma membrane‐bound TMEM16F can be seen as a sensor for PM integrity. It detects the breaches in the PM by locally elevated Ca^2+^. This role might represent a primitive form of cellular self‐protection, enabling cells to defend their viability by keeping the PM sealed against various environmental assaults. However, given its extensive modulation of PM lipids, TMEM16F might have more profound influences on cell survival and signaling than typical danger sensors.

The innate immune response serves not only as the first line of defense against bacterial infections but also plays a critical role in bridging the transition to adaptive immunity. A well‐coordinated innate immune response efficiently controls the early stage of infection by recruiting monocytes and neutrophils to infected foci, thereby limiting inflammation, as demonstrated in our data. Furthermore, during *Lm* infection, innate immune cells participate in tissue damage repair by interaction with structural cells, such as hepatocytes in the liver. In cases of unrestrained infection, as observed in TMEM16F‐deficient mice, innate immune cells fail to contain bacteria propagation. The resulting PM rupture of infected cells leads to the release of intracellular contents, provoking excessive inflammation and dysregulated metabolism, which ultimately causes severe tissue damage, even death. While low‐grade *Listeria* infection is typical self‐limiting, its impact on adaptive immunity varies. Myeloid cells are critical antigen‐presenting cells (APCs). They capture antigens from bacteria, process them, and then present them to T cells. Lysis of *Listeria*‐bearing APCs in the absence of TMEM16F would impair the antigen presentation. Beyond the loss of KCs in numbers, altered membrane markers and dysregulated metabolisms would harm the transition from innate to adaptive anti‐bacterial immunity. Besides, metabolic status and monocyte trafficking could also influence adaptive immunity to bacterial infection.^[^ ^56^, ^70^ ^]^ Given that hepatocytes are the principal cells in the liver and we did not see altered bacterial loads in WT and Alb‐Cre TMEM16F KO mice, we believe that the changes in lipids observed in our liver lipidome results are likely due to dysregulated metabolism indirectly elicited by excessive inflammation triggered by KCs death. Further studies are warranted to clarify the detailed mechanisms of inflammation, metabolism, and the formation of effective adaptive immunity upon bacterial infection.

## 4 Experimental Section

### Mice

All mice used in this study were on the C57BL/6J genetic background. Sex‐matched mice aged 6–12 weeks were used for the experiments. The TMEM16F germline KO mice were previously described.^[^ ^30^ ^]^ If not explicitly mentioned in the text, *Tmem16* *f* ^+/+^ littermates were used as controls for germline KO mice. To get the tissue‐specific deletion of TMEM16F, *Tmem16f* floxed mice were crossed with various Cre‐expressing mice, including LysM‐iCre, Cd4‐Cre, Cd19‐Cre or Alb‐cre. The Cd4‐Cre mice were kind gifts from Professor André Veillette of Institut de Recherches Cliniques de Montréal (IRCM). *Cx3cr1‐Cre* and tdTomato reporter mice were kindly provided by Professor Shuo Yang at Nanjing Medical University. B6/JGpt‐Albem1Cin(IRES‐iCre)/Gpt (Strain NO: T055035) and B6/JGpt‐Lyz2em1Cin(iCre)/Gpt (Strain No: T003822) mice were from GemPharmatech (Nanjing, China). B6.129P2(C)‐Cd19tm1(cre)Cgn/J (Strain No: 006785) was from Jackson laboratory. *Tmem16f‐ZsGreen* knock‐in mice were made by CRISPR‐Cas9 technology (GemPharmatech). Briefly, the P2A‐*ZsGreen* DNA cassette was inserted right after the last exon of *Tmem16f* on chromosome 15 (See the detailed strategy in Figure S3A, Supporting Information). The *Tmem16f‐ZsGreen* reporter mice have normal TMEM16F functions and breed normally. Both females and males were used for the studies. Mice were maintained in specific pathogen‐free (SPF) animal facilities. All animal experimentation was approved by the Animal Care Committee of Huazhong University of Science and Technology (Approval No. 3814).

### Antibodies, Reagents, and Cell Lines

Rabbit anti‐mouse TMEM16F monoclonal antibody was developed and produced by Abcam (Cat. ab234422). Antibodies against mouse CD4 (GK1.5), CD8α (53‐6.7), TCRβ (H57‐597), NK1.1 (PK136), CD19 (6D5), B220 (RA3‐6B2), Ly6G (1A8), Gr‐1 (RB6‐8C5), Ly6C (AL‐21), CD11b (M1/70), F4/80 (BM8), CD45 (30‐F11), CD11c (N418), TIM‐4 (RMT4‐54), CD64 (X54‐5/7.1), CD36 (HM36), CLEC‐2 (17D9/CLEC‐2), Ki‐67 (16A8), CD206 (C068C2), Annexin V (Cat.640941) and matched isotypes were from BioLegend (San Diego, CA). ESAM Monoclonal Antibody (1G8) was from eBioscience. VSIG4 (NLA14) monoclonal antibody was purchased from Invitrogen (San Diego, CA). β‐actin monoclonal antibody (Cat. 66009‐1‐Ig) was from Proteintech (Wuhan, China). Inhibitors used in the experiments: Necrostatin 2 (Cat. A3652, APExBIO), Ferrostatin‐1 (Cat. A4371, APExBIO), Z‐VAD‐FMK (Cat. A1902, APExBIO) and Ac‐YVAD‐CMK (Cat. C4810, APExBIO), T16A(inh)‐A01 (Cat.18518, Cayman). RMA cells were previously described^[^ ^71^, ^72^ ^]^ and the RMA TMEM16F KO cells were generated by CRISPR‐Cas9‐mediated gene editing. The RMA TMEM16F KO rescued cells were generated by lentiviral constructions. Immortalized BMDM (iBMDM) cells were kindly provided by Dr Xing Liu (Institut Pasteur of Shanghai).

### Bacteria, Plasmids, cDNAs

The *L. monocytogenes* (10403s) was kindly provided by Professor Dan Portnoy of UC California, Berkeley. The *L. monocytogenes* GFP and the Δ*actA* and Δ*hly* strains were kind gifts from Professor Grégoire Lauvau at Albert Einstein College of Medicine. All the strains were grown at 37 °C in brain heart infusion (BHI) medium. Mouse cDNAs of *Tmem16f* and *Tmem16a* were obtained from OriGene. pSpCas9‐2A‐GFP (px458, #48138) plasmids were from Addgene. The TMEM16A‐SCRD chimeras were cloned as described previously.^[^ ^49^ ^]^ RMA cells were nucleofected with the Cell Line Nucleofector Kit R (Lonza, Cat. VCA‐1001) following the manufacturer's instructions.

### Listeria Monocytogenes Infection in Vivo and in Vitro

Infection of mice with *Lm* was performed under Biosafety Level 2 practices and containments. Briefly, *Lm* was grown overnight in BHI medium containing 200 µg mL^−1^ of streptomycin. Bacterial culture was boosted in fresh growth medium for two hours at 37 °C. Bacteria in the exponential growth phase [optical density (OD)_600nm_ = 0.18 to 0.25] were then collected and enumerated using the following formula: 1 OD_600 nm_ = 0.7×10^9^ CFU mL^−1^. After washing in PBS, bacteria were resuspended at the desired concentration, and 200 µL of bacteria suspension was injected intravenously.

Mice were euthanized at the days indicated post‐infection, and liver and spleen were isolated and homogenized, followed by serial dilution in PBS and plated onto BHI agar plates containing streptomycin to count the colonies after 24 h of incubation at 37 °C. Serum IL‐18 was measured by ELISA according to the manufacturer's protocol (Cat. 88‐50618‐88, Invitrogen). In addition, serum alanine aminotransferase (Cat. H001A) and aspartate aminotransferase (Cat. H002A) activity was analyzed by following the product manual from the Medical System Biotechnology Company. Data were collected with the Beckman Coulter Chemistry Analyzer System (AU480) according to the Alanine Substrate Method and Aspartate Substrate Method.

Frozen liver sections were stained with Oil Red O (Cat. O0625, Sigma) to detect neutral lipids. Slides were scanned with Olympus Virtual Slide Microscope (VS120‐S6‐W). The Oil Red O images were quantified using the ImageJ software.

For in vitro infection of cells with *Lm*, half million cells were co‐cultured with bacteria at indicated MOI in RPMI‐1640 medium containing 10% fetal bovine serum but with no gentamicin in 96‐well U‐bottom plates. The cell suspension was mixed and centrifuged at room temperature, 800 rpm for 1 min, then gently transferred to the cell incubator. Cells were then collected at the indicated time after incubation and stained for subsequent assay. For the detection of Annexin V/PI, cells were pelleted and suspended in 1x binding buffer (eBioscience) containing Annexin V (5 ng mL^−1^) and propidium iodide (100 ng mL^−1^, Cat. P4170, Sigma), or DAPI (100 nm, Cat. 422801, BioLegend), followed by the analysis on the LSRFortessa flow cytometer (BD) without wash. To assess membrane lipid fluidity and rigidity, cells were stained with Merocyanine 540 (Cat. 323756, Sigma) and Laurdan dye (Cat. HY‐D0080, MedChemExpress) for 30 min at 37 °C followed by *Lm* infection for two hours. For the detection of leakage of intracellular contents after *Lm* infection, thymocytes stained with 5(6)‐Carboxyfluorescein diacetate N‐succinimidyl ester (CFSE, Cat. C34554, Thermo), were then inspected by flow cytometry.

### Lactate Dehydrogenase (LDH) Release Cell Death Assay

Cells were plated in a 96‐well plate with *Lm* infection. After incubation, the supernatant was collected for LDH detection by the LDH cytotoxicity assay kit (Cat. C0017, Beyotime) according to the manufacturer's instruction. Absorbance at 490 nm was measured on the Synergy H1 microplate reader (BioTek). The cell death ratio was calculated using the formula: Cell death ratio (%) = (A_sample_‐A_control_/A_max_‐A_control_) ×100%. A_sample_ represents the sample absorbance value, A_control_ represents the absorbance value of the control group, and A_max_ represents the absorbance value of the maximal release group.

### Intravital Imaging of Kupffer Cells During *L. Monocytogenes* Infection

The intravital microscopy of Kupffer cells during *Lm* infection was previously established.^[^ ^73^ ^]^ Briefly, for in vivo imaging of *Lm* WT GFP capturing in the liver, Kupffer cells were visualized by intravenous injection of fluorescence‐conjugated anti‐F4/80 immediately prior to liver preparation. 5×10^7^ CFU *Lm* WT GFP was injected at the beginning of imaging, and five fields were recorded for 20 min in parallel. The dynamics of Kupffer cell death were imaged after *Lm* WT GFP infection with 1×10^8^ CFU for 1 h, followed by injecting 25 µg Propidium Iodide (PI). To visualize the *Lm* WT GFP escaping from Kupffer cells, mice were infected with 5×10^7^ CFU bacteria for 12 h, and Kupffer cells were labeled with anti‐F4/80 and anti‐TIM4. 5–10 fields were recorded for each mouse and analyzed by NIS‐Elements AR‐SP software (Nikon).

### Flow Cytometry Analysis

Single‐cell suspensions were isolated from the thymus, spleen, liver and bone marrow. After the red blood cell lysis, thymocytes (1×10^6^), bone marrow cells (5×10^6^), splenocytes (5×10^6^) and percoll‐enriched liver immune cells (5×10^5^) were stained with antibodies dilutions in PBS containing 2% FBS and mouse Fc blockers for 30 min on ice for cell surface staining. To detect the cells infected by *Listeria*, mice were infected with 5×10^8^ CFU GFP‐expressing *Lm* for 6 h. The GFP^+^ immune cell subsets from the thymus, spleen, liver, and bone marrow were identified. DAPI was added in a concentration of 100 nm in 300µL PBS containing 2% FBS for viability staining. For intracellular staining, cells were first stained with surface antibodies and washed with 1 mL PBS containing 2% FBS. Subsequently, the cells were fixed and permeabilized with Fixation/Permeabilization Solution (Cat. 51–2090kz, BD Biosciences) and stained with anti‐Ki‐67 in Perm/Wash buffer (Cat. 51–2091kz, BD Biosciences).

Unless specified, for lipid scrambling evaluation in vitro, cells infected with *Lm* at indicated MOI and time were directly labeled with Annexin V (5 ng mL^−1^) and 100 ng mL^−1^ PI (or 100 nm DAPI). For monitoring the lipid scrambling from the outer to the inner leaflet of PM, NBD‐PC (Cat. 810132P, Sigma) and NBD‐Sphingomyelin (Cat. 810218P, Sigma) were used for staining cells. Subsequently, equal volume of cell suspension and the prechilled RPMI‐1640 containing 5 mg mL^−1^ BSA was mixed to extract the unincorporated fluorescent lipids. DAPI was added to exclude the dead cells. PE‐binding probe duramycin (MOLECULAR TARGETING TECHNOLOGIES, PA) was used to detect PE exposure during lipid scrambling. Stained samples were subjected to flow cytometry analysis on an LSRFortessa flow cytometer (BD). All the data were analyzed by FlowJo software V10.

### Kupffer Cell Isolation

For Kupffer cell isolation, mice were anesthetized, and the hepatic portal vein was cannulated under aseptic conditions. The liver was subsequently perfused with an EGTA solution (0.19 g L^−1^) and digested with a solution containing CaCl_2_ (0.05%), Pronase E (0.025%, Roche), collagenase (0.025%, Sigma) and DNase I (10 µg mL^−1^, Worthington). Next, the liver was cut into ≈2 mm^3^ pieces and shaken for 20 min at 100 rpm in a 37 °C incubator. The digested mixture was centrifuged at 50 g for 5 min to remove liver parenchymal cells. Finally, the solution was centrifuged at room temperature at 500 g for 8 min. The pellet was washed with PBS and re‐suspended with 3 mL 20% percoll (Cat. 17‐0891‐01, GE Healthcare) and gently overlaid on 2 mL 50% percoll. Cells were centrifuged at 800 g for 20 min at 4 °C, and Kupffer cells were collected from the interphase. After twice washing with PBS, cells were re‐suspended in RPMI‐1640 medium for surface marker staining and flow cytometry analysis.

### Lipid Scrambling Induction by Ionomycin During Bacteria Infection

Thymocytes single‐cell suspensions were isolated from the WT mice thymus. After washing with PBS, 1×10^6^ thymocytes were added 100 µL RPMI‐1640 medium containing 10% fetal bovine serum and 2µM ionomycin in 96‐well U‐bottom plates. Next, 1×10^7^ CFU *Lm* (100 µL, MOI = 10) was added to the *Lm*‐treated well and the same volume RPMI‐1640 medium containing 10% fetal bovine serum was added to the control well immediately. The cell suspension was mixed and centrifuged at room temperature, 800 rpm for 1 min, then gently transferred to the cell incubator. After incubation for 1h, cells were pelleted and suspended in 1x binding buffer (eBioscience) containing Annexin V (5 ng mL^−1^,) and propidium iodide (100 ng mL^−1^, Cat. P4170, Sigma), followed by the analysis on the LSRFortessa flow cytometer (BD) without wash.

### Bulk RNA‐Sequencing

WT and TMEM16F KO mice at 8 weeks old were infected with 1×10^4^ CFU of *Lm* intravenously for 3 days. The mice were anesthetized at 3 dpi and the liver was perfused with sterile PBS to remove blood. ≈0.1 g of liver tissue was excised and rapidly immersed in liquid nitrogen for quick‐freezing. The tissue was then placed into an enzyme‐free Eppendorf tube and immediately stored at −80 °C for subsequent RNA extraction. Total RNA from the liver tissues of WT and TMEM16F KO mice was extracted using Trizol (Invitrogen, Carlsbad, CA). Subsequently, RNA samples were quantified and qualified using a NanoDrop and Agilent 2100 bioanalyzer (Thermo Fisher Scientific, MA). mRNA library was constructed and sequenced on the Illumina BGISEQ500 platform by BGI‐Shenzhen. The sequencing data were filtered with SOAPnuke (v1.5.2), and the clean reads were mapped to the GCF_000001635.26_GRCm38.p6 genome sequence from NCBI using HISAT2 (v2.0.4). Bowtie2 (v2.2.5) was applied to align the clean reads to the reference coding gene set, then the expression level of the gene was calculated by RSEM (v1.2.12). Essentially, DEseq2 algorithms were used to identify the differentially expressed genes (DEGs). Genes with absolute log2‐transformed fold changes >1 and a threshold of p‐value <0.05 were considered as DEGs. GO and GSEA analysis of DEGs was performed by clusterProfiler80 R package, and heatmaps were generated using the pheatmap R package (https://github.com/raivokolde/pheatmap). Kyoto Encyclopedia of Genes and Genomes (KEGG) pathway enrichment analysis was conducted using ClusterProfiler (v3.18.1). Significantly enriched KEGG pathways were identified if their adjusted p‐value was <0.05. The p‐values of the DEGs were obtained via the Wilcoxon rank‐sum test. The p‐value was obtained using a Kolmogorov‐Smirnov‐like statistic for GSEA. Subsequently, the p‐values were corrected using the Benjamini‐Hochberg (BH) method to obtain adjusted p‐values.

### Lipidomics

For the liver samples in lipidomic analysis, liver tissues were obtained from 8‐week‐old WT and TMEM16F KO mice infected with 1×10^4^ CFU *Lm* for 3 days. At 3 dpi, the mice were anesthetized, and the liver was perfused with sterile PBS to remove blood. A portion of liver tissue was excised and immersed in liquid nitrogen for quick‐freezing, and then placed into −80 °C pre‐cooled Eppendorf tube for liver lipidomic analysis by LipidALL Technologies. The plasma membrane from thymocytes was purified using hypotonic solutions. Briefly, thymocytes were treated in Annexin V binding buffer containing 1 nm LLO or 5 µm ionomycin for 3 min at room temperature, and cells were pelleted at 500 g for 3 min. The cells were then suspended with 800 µL membrane extraction buffer (210 mm mannose, 70 mm sucrose, 5 mm Tris, 1 mM EDTA, 1 mM PMSF (Cat. 10837091001, Sigma), 6.25 µg mL^−1^ BHT (Cat. 47168, Sigma), 6.25 µg mL^−1^ BHA (Cat. PHR1306, Sigma) and removed to a 2 mL glass homogenizer for grinding ≈250 times on the ice. The liquid was then completely transferred to a new tube and centrifuged at 1000 g for 10 min at 4 °C. Next, the supernatant was removed to a new tube and was centrifuged at 3500 g for 15min at 4 °C. The pellet was washed by the membrane extraction buffer and subsequently sent for lipidomic analysis by LipidALL Technologies.

Lipids were extracted from ≈50 mg of frozen tissues or purified cellular membranes using a modified version of the Bligh and Dyer's method described previously.^[^ ^74^ ^]^ Briefly, tissues were homogenized in 750 µL of chloroform: methanol: MilliQ H2O (3:6:1) containing 1% (w/v) butylated hydroxytoluene (v/v/v). The homogenate was then incubated at 1500 rpm for 1 h at 4 °C. At the end of the incubation, 350 µL of deionized water and 250 µL of chloroform were added to induce phase separation. The samples were then centrifuged, and the lower organic phase containing lipids was transferred into a clean tube. Lipid extraction was repeated once by adding 450 µL of chloroform to the remaining aqueous phase, and the lipid extracts were pooled into a single tube and dried in the SpeedVac under OH mode. Samples were stored at ‐80 °C until further analysis. Lipidomic analyses were conducted at LipidALL Technologies using an Exion LC‐AD coupled with Sciex QTRAP 6500 PLUS, as reported previously.^[^ ^75^ ^]^ Separation of individual lipid classes of polar lipids by normal phase‐HPLC was carried out using a TUP‐HB silica column (i.d. 150×2.1 mm, 3 µm) with the following conditions: mobile phase A (chloroform: methanol: ammonium hydroxide, 89.5:10:0.5) and mobile phase B (chloroform: methanol: ammonium hydroxide: water, 55:39:0.5:5.5). MRM transitions were set up for comparative analysis of various polar lipids. Individual lipid species were quantified by referencing to spiked internal standards. DMPC, DMPE, DMPG, d31‐PS, diC8‐PI, C17‐PA, C14‐BMP, C17‐LPC, C17‐LPE, C17 LPA, C17‐LPS, C17‐LPI, C17:1‐LPG, CL80:4, C12‐SM, Cer(d18:1‐d7/15:0), C8‐GluCer, C8‐GalCer, LacCer‐d3(18:1/16:0), d17:1‐S1P, d17:1‐Sph,C17 Gb3, C17‐SL, d3‐16:0‐carnitine were obtained from Avanti Polar Lipids. GM3‐d18:1/18:0‐d3 was purchased from Matreya LLC. Free fatty acids were quantitated using d31‐16:0 (Sigma‐Aldrich) and d8‐20:4 (Cayman Chemicals). Glycerol lipids, including diacylglycerols (DAG) and triacylglycerols (TAG) were quantified using a modified version of reverse‐phase HPLC/MRM. Separation of neutral lipids was achieved on a Phenomenex Kinetex‐C18 column (i.d. 4.6×100 mm, 2.6 µm) using an isocratic mobile phase containing chloroform:methanol:0.1 m ammonium acetate 100:100:4 (v/v/v) at a flow rate of 300 mL for 10 min. Levels of short‐, medium‐, and long‐chain TAGs were calculated by referencing to spiked internal standards of TAG (14:0)3‐d5, TAG (16:0)3‐d5, and TAG(18:0)3‐d5 obtained from CDN isotopes, respectively. DAGs were quantified using d5‐DAG17:0/17:0 and d5‐DAG18:1/18:1 as internal standards (Avanti Polar Lipids). Free cholesterols and cholesteryl esters were analyzed under atmospheric pressure chemical ionization (APCI) mode on a Jasper HPLC coupled to Sciex 4500 MD as described previously, using d6‐cholesterol and d6‐C18:0 cholesteryl ester (CE) (CDN isotopes) as internal standards. Other methodological details, including MRM transitions, were comprehensively reported in a preceding publication.^[^ ^76^ ^]^

For the analysis of lipidomic data, differences in lipid levels between WT and TMEM16F KO groups were assessed using an unpaired Student's *t*‐test with Benjamini–Hochberg correction. A p‐value of <0.05 was considered statistically significant for all comparisons. The circular bar plot and volcano plot were created using the ggplot2 R package. The resulting p‐values were adjusted using the Benjamini‐Hochberg method to control the false discovery rate (FDR). Significant differentially expressed lipids were defined as having a *p*‐value <0.05 in the circular bar plot and a *p*‐value <0.05 with FC>1.2 or FC<1/1.2 in the volcano plot. The heatmap was generated using the pheatmap package in R after data standardization. The color bar on the right side of the heatmap indicates the value corresponding to each color, representing the number of standard deviations from the mean.

### Western Blot

Cells were lysed in RIPA buffer supplemented with proteinase and phosphatase inhibitor cocktails. Proteins were quantified using BCA methods, and denatured by boiling for 5 min in 1x sampling buffer, followed by separation in SDS‐PAGE. Proteins on the gel were transferred to PVDF membranes and incubated with indicated primary antibodies for 1.5 h at RT and then with horseradish peroxidase (HRP) conjugated secondary antibodies for 1 h. The chemiluminescence was detected on ChemiDoc XRS+ Imaging System (Bio‐Rad).

### Statistical Analysis

GraphPad Prism software V8 was used for the plots and statistical analysis (GraphPad Software Inc.). No data were excluded. All the experiments were performed at least two times independently, and the statistical analysis details are available in the figure legends. N depicts the number of animals used, the number of technical replicates, or the number of pictures quantified, as specified in the legends. Data are means ± sem. P values less than 0.05 were considered to be statistically significant.

## Conflict of Interest

The authors declare no conflict of interest.

## Supporting information

Supporting Information

Supplemental Video 1

Supplemental Video 2

## Data Availability

The data that support the findings of this study are available from the corresponding author upon reasonable request.
