# Supplementary material for: TMEM16F Expressed in Kupffer Cells Regulates Liver Inflammation and Metabolism to Protect Against Listeria Monocytogenes
Source: Adv Sci (Weinh). 2024 Aug 13;11(39):2402693. doi: 10.1002/advs.202402693 (PMC11497084; doi:10.1002/advs.202402693)
Supplement: Supplementary file 1 — Supporting Information [file ADVS-11-2402693-s001.docx]

Supporting Information

**TMEM16F expressed in Kupffer cells regulates liver inflammation and metabolism to protect against *Listeria monocytogenes***

Jianlong Tang, Hua Song^#^, Shimin Li^#^, Sin Man Lam, Jieming Ping, Mengyun Yang, Na Li, Teding Chang, Ze Yu, Weixiang Liu, Yan Lu, Min Zhu, Zhaohui Tang, Zheng Liu, Yusong R. Guo, Guanghou Shui^*^, André Veillette^*^, Zhutian Zeng^*^, and Ning Wu^*^

*^#^These authors contributed equally to this work*

**Supplementary figures**


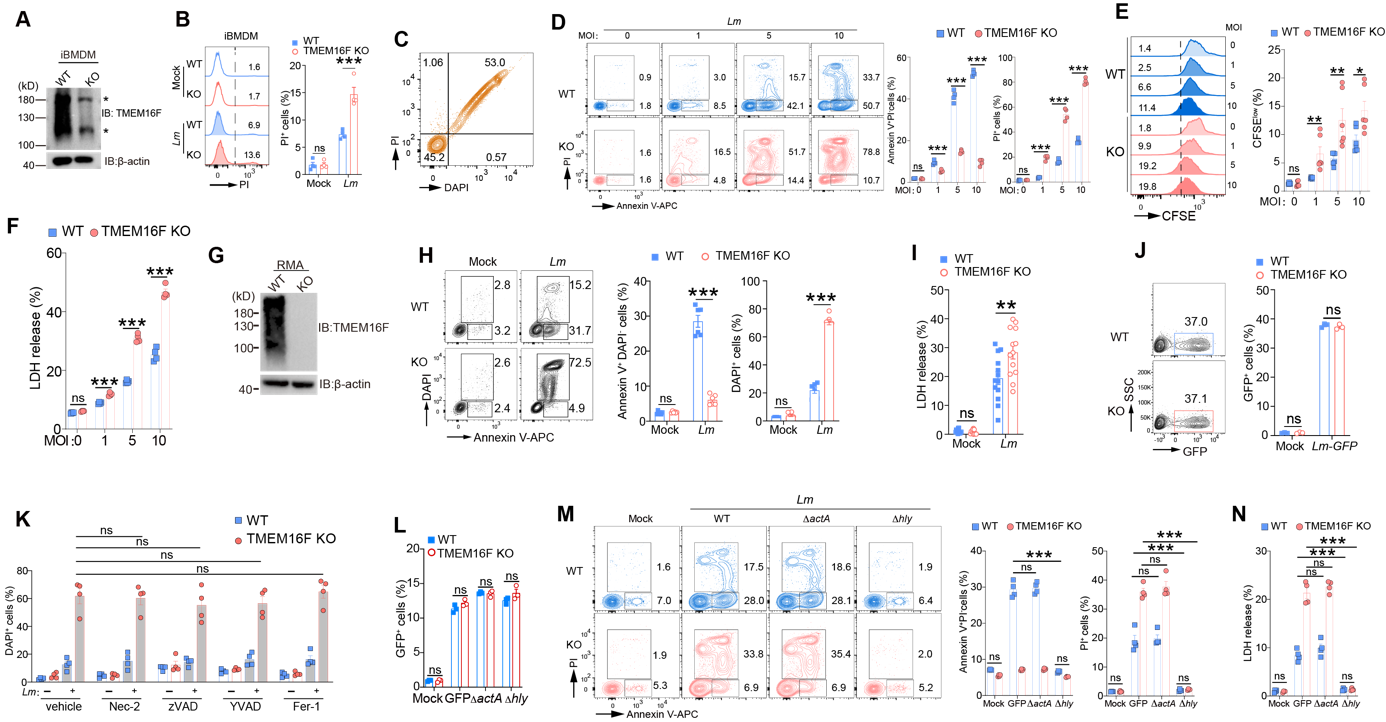


**Fig. S1.** **TMEM16F mediates PS exposure and counteracts cell death induced by *Lm* infection.** (A) Immunoblot of TMEM16F in WT and TMEM16F KO immortalized BMDM (iBMDM) cells. *: non-specific band. (B) Representative histograms (left) and quantification (right) of PI staining in iBMDM cells infected with *Lm* for 9h. MOI=15, n=4. (C) Representative flow cytometry plots of PI (100 ng/ml) and DAPI (100nM) staining for dead cells after *Lm* infected TMEM16F KO RMA cells. (D) Flow cytometry analysis of lipid scrambling (shown by PS exposure detected by Annexin V staining) and death (shown by PI^+^) in thymocytes (1x 10^6^ cells) after *Lm* infection for 2h at the indicated MOI. Quantification of PS exposure (Annexin V^+^PI^-^) and cell death (PI^+^) on the right panel (n=4). (E) Representative flow cytometry plots (left) and statistics (right) of CFSE leakage (CFSE^low^) from thymocytes infected by *Lm* as in D. (n=6). (F) LDH release measured for thymocytes infected by *Lm* as in D*.* (n=4). (G) Immunoblot of TMEM16F in WT and TMEM16F KO RMA cells. (H) Representative flow cytometry plots (left) and frequencies of PS exposure and cell death (right) of RMA cells after *Lm* infection for 2h. MOI=15, n=6. (I) LDH assay of RMA cells after *Lm* infection for 2h. MOI=15, n=13. (J) Representative flow cytometry plots (left) and frequencies of *Lm-*GFP infection (right) in WT and TMEM16F KO RMA cells. MOI=15, n=3. (K) Quantification of cell death of RMA cells incubated with indicated inhibitors and *Lm* for 2h. MOI=15, n=4. (L) Percentage of GFP-expressing WT, actA KO (Δ*actA*) and LLO KO (Δ*hly*) *Lm* infection in WT and TMEM16F KO thymocytes for 2h *in vitro*. MOI=5, n=3. (M) Flow cytometry analysis of PS exposure and cell death in thymocytes after WT, actA KO (Δ*actA*) and LLO KO (Δ*hly*) *Lm* infection as in (L). Statistical analysis of PS exposure and cell death (right panel, n=4). (N) LDH release evaluated on the same experiment as panel (M). All the experiments above were repeated at least twice independently and data are presented as mean ± SEM. The “n” represents the technical replicates. Statistical analysis by two-way ANOVA for panels B, D to F, and H to N. ns, not significant; *p< 0.05, **p< 0.01, ***p< 0.001.


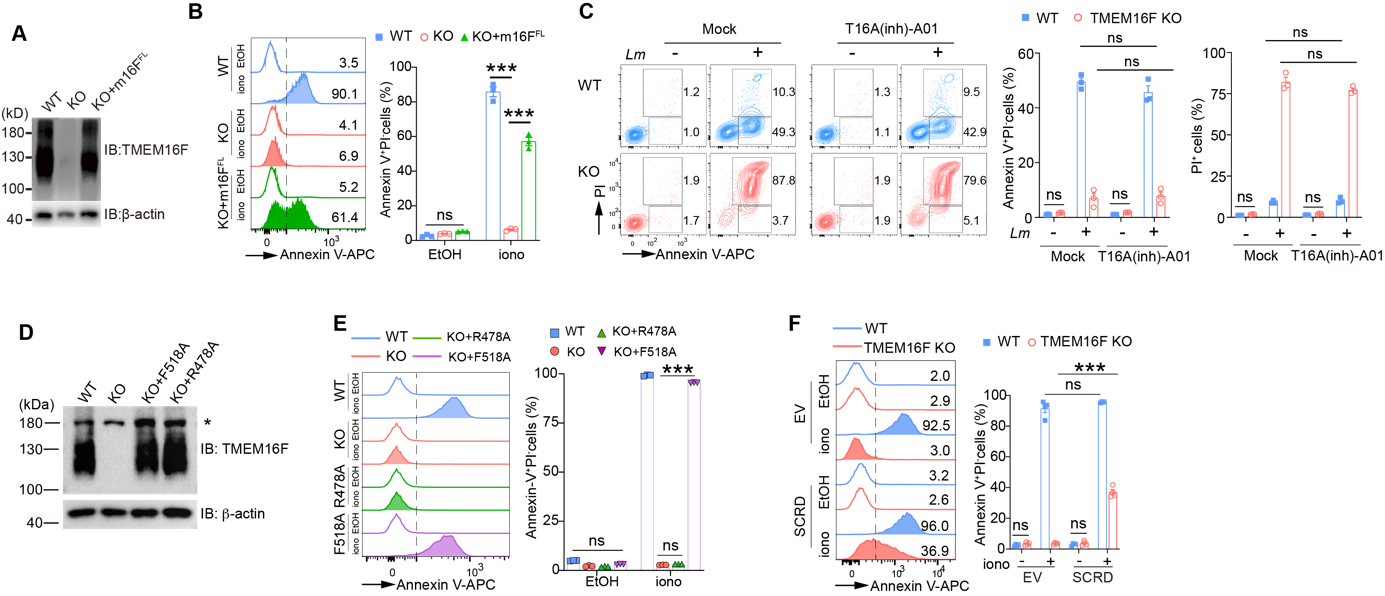


**Fig. S2.** **Lipid scrambling by TMEM16F essentially contributes to the resistance to PM permeabilization induced by *Lm*.** (A) Immunoblot of mouse TMEM16F in WT, TMEM16F KO and KO rescued with full length TMEM16F (KO+m16F^FL^) RMA cells. β-actin as the loading control. (B) Representative histogram (left) and frequencies (right) of PS exposure in RMA cells after ionomycin (5μM) treatment. n=3. FL, full length. (C) Representative flow cytometry plots (left) and frequencies of PS exposure and cell death (right) of WT and TMEM16F KO RMA cells incubated with *Lm* and T16A(inh)-01 for 2h. n=3. (D) Immunoblot of WT, TMEM16F KO and KO overexpressing R478A, F518A RMA cells. *: non-specific band. (E) Representative histogram (left) and frequencies (right) of PS exposure in RMA cells expressing TMEM16F mutants after ionomycin (5μM) treatment. n=3. (F) Representative histogram (left) and frequencies (right) of PS exposure in RMA cells expressing scramblase domain chimeras (SCRD) after ionomycin (5μM) treatment. n=4. Experiments were performed for at least two independent times. Data are presented as mean ± SEM. The “n” represents the technical replicates. Statistical analysis by two-way ANOVA (B, C, E and F). ns, not significant; ***p< 0.001.


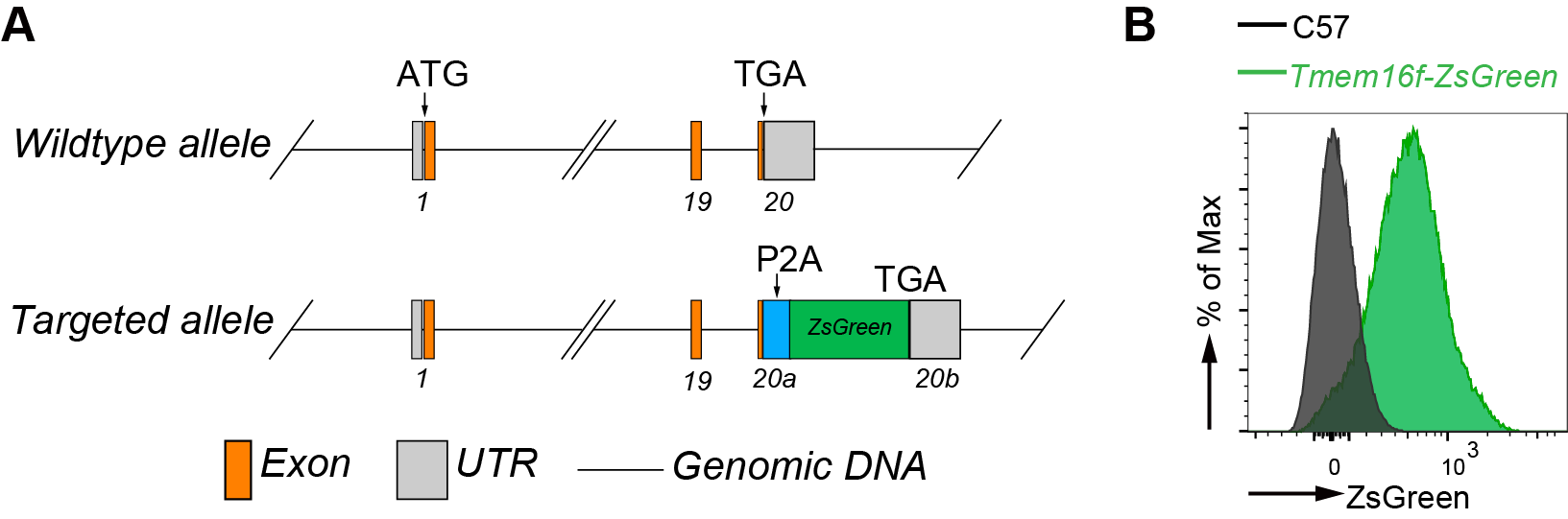


**Fig. S3. Lipid scrambling mediated by TMEM16F maintains plasma membrane integrity from injury by *Lm* infection.** (A) Schematic diagram for the strategy of generation of TMEM16F-ZsGreen reporter mice. (B) Representative histogram of ZsGreen level in the thymocytes from the TMEM16F-ZsGreen reporter mice. C57 as the negative control. Experiments were performed for at least two independent times.


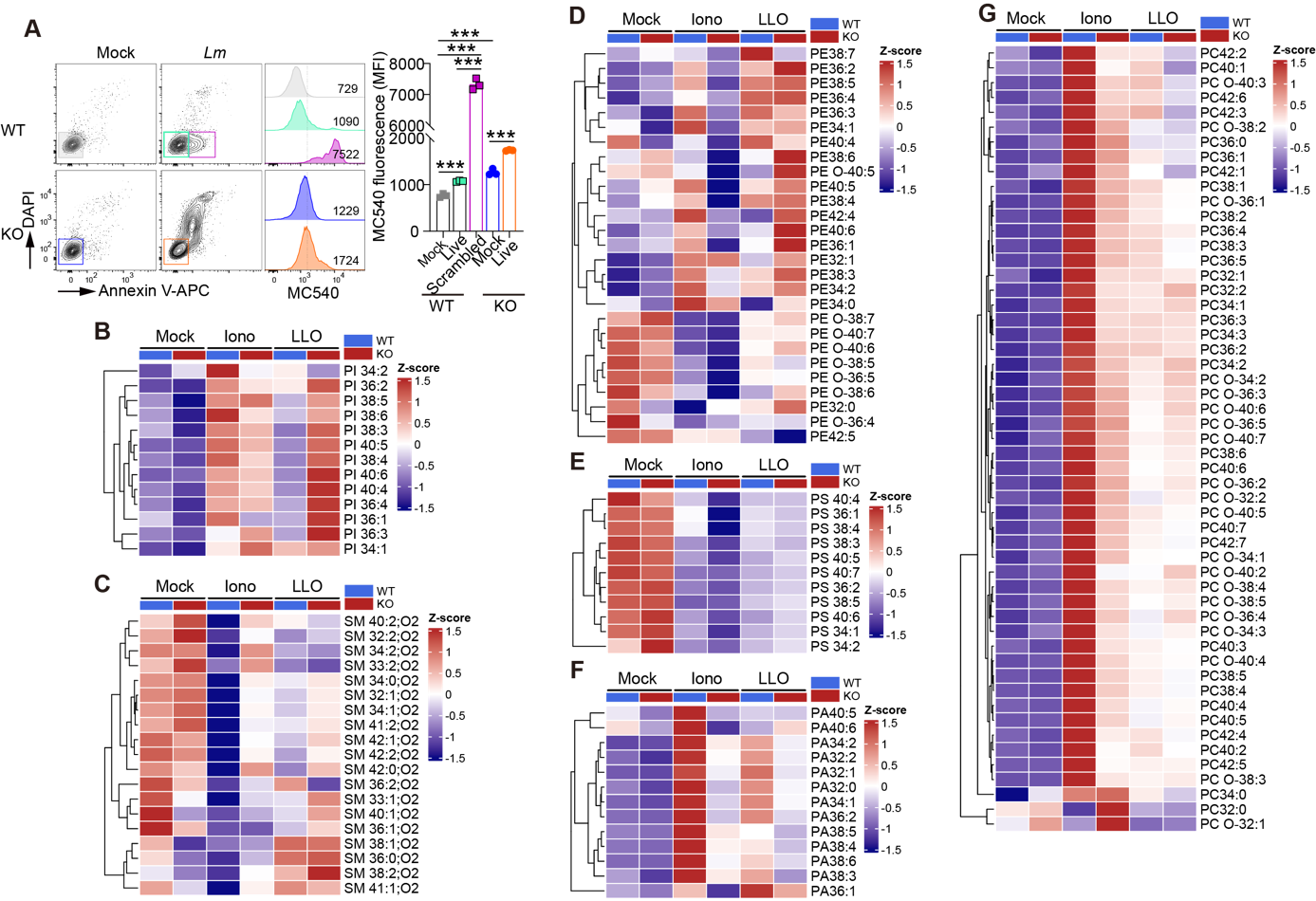


**Fig. S4. Extensive plasma membrane lipids re-modeling by TMEM16F during *Listeria* infection.** (A) Representative flow cytometry plots of Annexin V and DAPI (left) in RMA cells after *Lm* infection and the MFI of merocyanine 540 (MC540) (right) from the indicated gates. n=3. (B to G) The lipidomics of plasma membrane purified from thymocytes treated with ionomycin and LLO. PI, phosphatidylinositol; SM, sphingomyelin; PE, phosphatidylethanolamine; PS, phosphatidylserine; PA, phosphatidic acid; PC, phosphatidylcholine. Data are repeated at least twice and presented as mean ± SEM. The “n” represents the technical replicates. Statistical analysis by unpaired Student’s *t* test. ***p< 0.001.


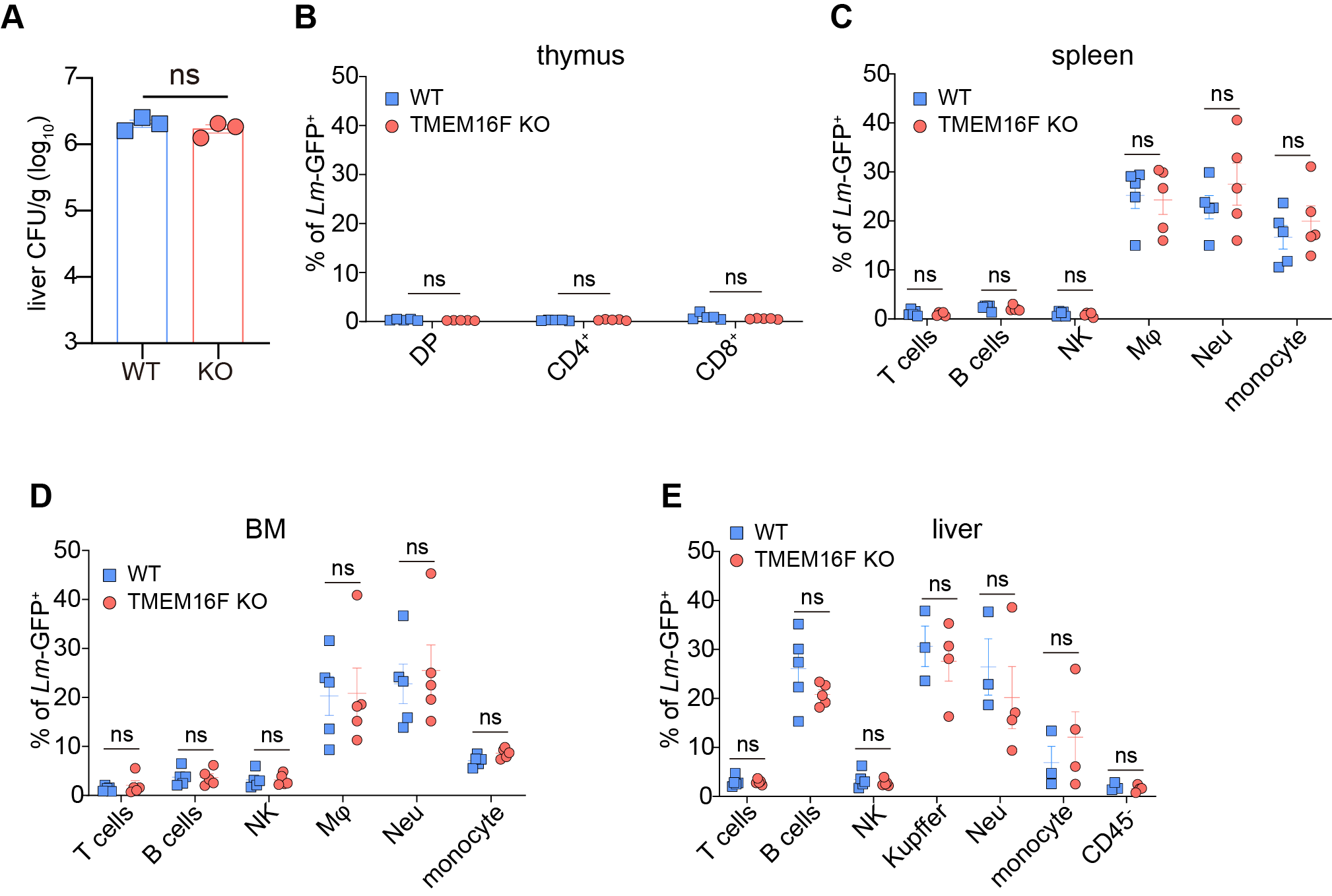


**Fig. S5. WT and TMEM16F KO mice are equally infected by *Lm*, but more severe hepatic injury in TMEM16F KO mice.** (A) Bacterial load in the liver from WT and TMEM16F KO mice after twenty minutes of *Lm* injection (5x10^7^ CFU) intravenously (n=3). (B to E) Immune cells harbors *Lm* intracellularly after GFP-expressing *Lm* infection (5x10^8^ number) for 6 hours in thymus (B), spleen (C), bone marrow (D), and liver (E). n=3-5. All the experiments were repeated at least twice. Data are pooled from at least three independent experiments (B to E) and presented as mean ± SEM. Statistical analysis by unpaired Student’s *t* test for B to E, two-tailed Mann-Whitney test for A. ns, not significant.


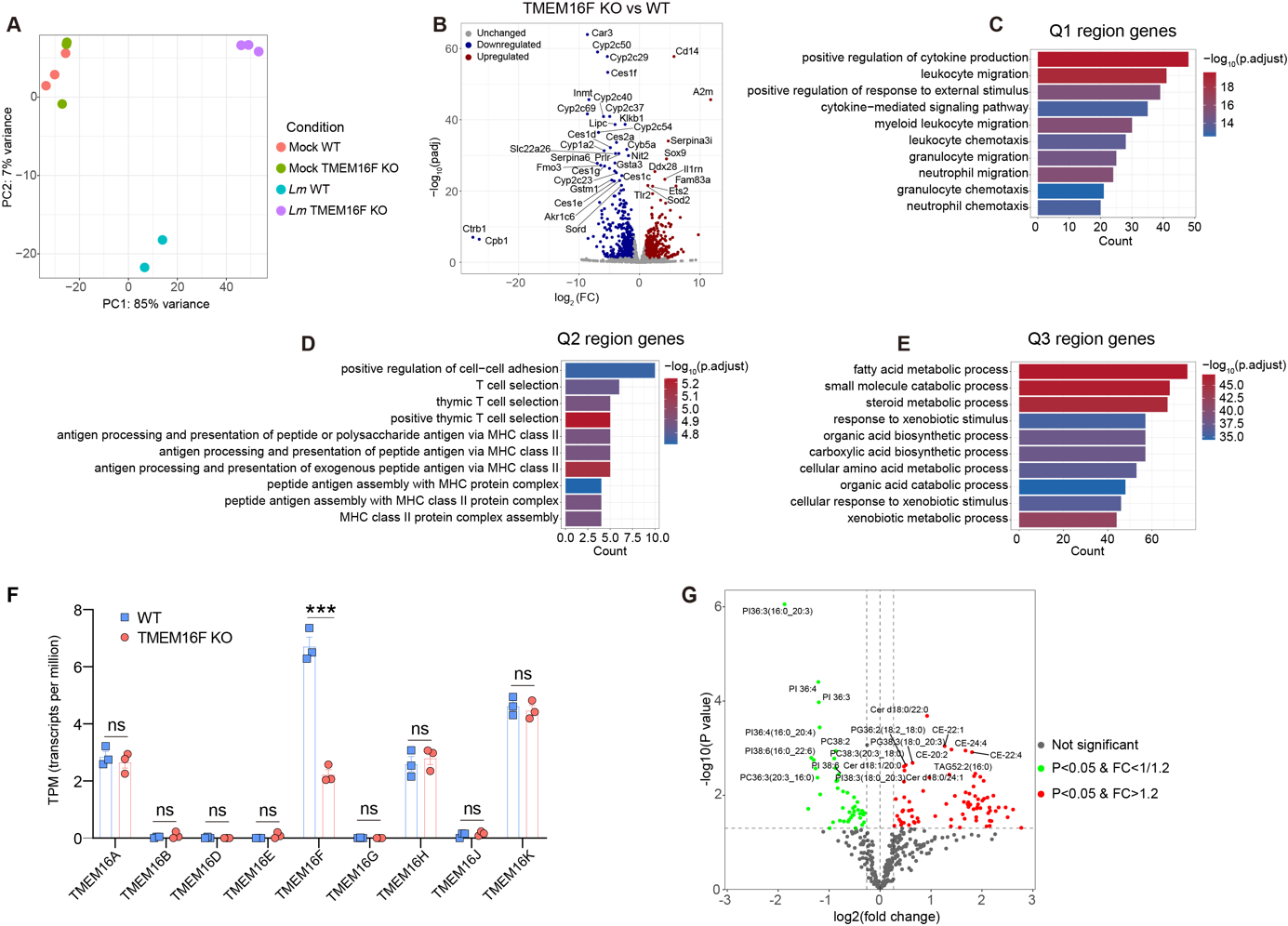


**Fig. S6. Proinflammatory response and aberrant cell metabolism in TMEM16F KO mice after *Lm* infection.** (A) Principal component analysis (PCA) of RNA-seq data from uninfected and *Lm-*infected WT and TMEM16F KO mice at 3 dpi. Dots represent individual samples. (B) Volcano plot of DEGs between WT and TMEM16F KO mice after *Lm* infection. Blue and red dots indicate downregulated and upregulated genes in TMEM16F KO mice, respectively. Genes with -log_10_p_adj_>20 and |log_2_FC|>20 were highlighted. (C to E) Top 10 KEGG pathways in Q1 (C), Q2 (D) and Q3 (E) regions displayed in the Fig. 5B. Color of the bar represents the negative logarithm p values. (F) The gene expression of TMEM16 family members in WT and TMEM16F KO mice liver. TPM, transcripts per million. n=3. (G) Volcano plot of lipids between WT and TMEM16F KO liver samples after *Lm* infection. Green and red dots indicate downregulated and upregulated genes in TMEM16F KO mice, respectively.


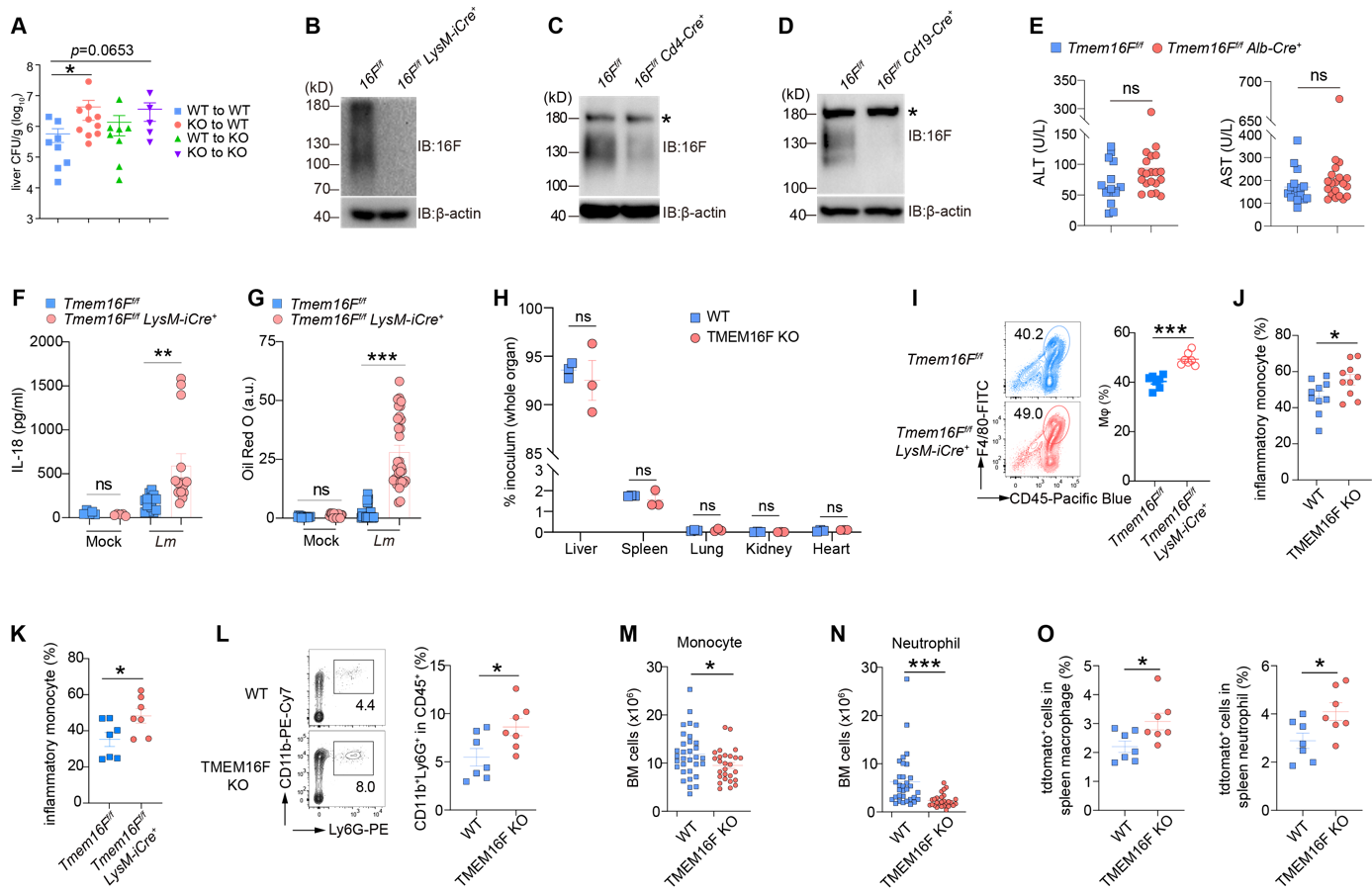


**Fig. S7. TMEM16F is required for myeloid cells in counteracting *Lm* infection.** (A) Liver bacterial load from BM reconstituted mice after *Lm* infection at 3 dpi. Data are pooled from two independent experiments. Each dot represents one mouse. (B to D) Immunoblot of various tissue-specific *Tmem16f* KO mice. Samples were BMDM (B), thymocytes (C), and splenic CD19^+^ B cells (D). β-actin is the loading control. *: non-specific band. (E) Quantification of serum ALT and AST activity from *Alb-Cre-Tmem16f^fl/fl^* and *Tmem16f^fl/fl^* control mice at 3 dpi. Data were pooled from two independent experiments. n=16-20. (F) Level of IL-18 in serum obtained from uninfected (Mock) and *Lm*-infected *LysM-iCre-Tmem16f^fl/fl^* and *Tmem16f^fl/fl^* control mice at 3 dpi. (n=5-14). (G) Quantification of Oil Red O staining of liver sections from uninfected (Mock) and *Lm*-infected *LysM-iCre-Tmem16f^fl/fl^* and control mice at 3 dpi. a.u., arbitrary unit. Mock, n=15; *Lm*, n=30. (H) WT and TMEM16F KO mice were infected i.v. with 1x10^4^ CFU of *Lm*. Bacterial load per organ were determined at 10 min post-infection and calculated as percentage of total inoculum. (I) Flow cytometry analysis of the liver cells in *Lm-*infected myeloid-specific *Tmem16f* KO mice at 3 dpi. Representative plots are on the left and statistical analysis of macrophages (CD45^+^F4/80^+^) is on the right (n=7). (J and K) Quantification of inflammatory monocytes (CD11b^int^Ly6C^hi^) in CD45^+^F4/80^+^ liver cells isolated from *Lm*-infected TMEM16F germline KO (J) and myeloid-specific KO (K) mice at 3 dpi (n=10 in J and 7 in K). (L) Flow cytometric analysis of liver neutrophils (CD45^+^CD11b^+^Ly6G^+^) from *Lm*-infected WT and TMEM16F KO mice at 3 dpi. Data are pooled from two independent experiments and quantified on the right panel (n=7). (M and N) Quantification of numbers of BM monocytes (CD45^+^CD11b^+^Gr-1^+^Ly6C^+^) and neutrophils (CD45^+^CD11b^+^Ly6G^+^), (n=27-32). (O) Quantification of splenic *Cx3cr1*-tdTomato^+^ macrophages and neutrophils transferred into WT and TMEM16F KO mice after *Lm* infection (n=7). Data are pooled from two independent experiments. Data are presented as mean ± SEM. Each dot represents one mouse (A, E-F, H-O) or a field of view (G). Statistical analysis by unpaired Student’s *t* test (I-L and O), one-way ANOVA (F and G) or two-tailed Mann-Whitney test (A, E, H, M and N). ns, not significant; *p< 0.05, **p< 0.01, ***p< 0.001.


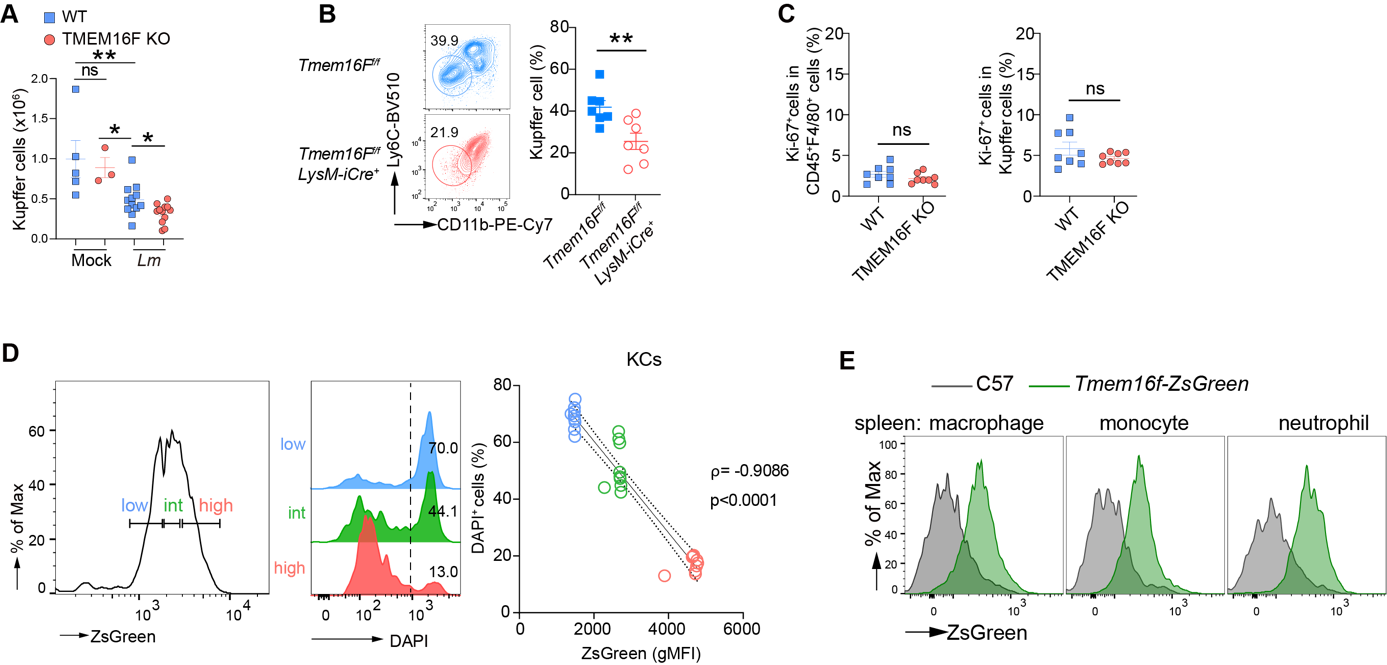


**Fig. S8.** **KCs with higher TMEM16F expression exhibited more resistant to *Lm*-induced death**(A) Quantification of numbers of Kupffer cells (CD45^+^F4/80^+^CD11b^lo^Ly6C^lo^) from *Lm*-infected TMEM16F germline KO mice at 3 dpi, (n=3-12). (B) Flow cytometry analysis of KCs on gated CD45^+^F4/80^+^ liver cells isolated from *Lm*-infected myeloid-specific *Tmem16f* KO mice at 3 dpi. KCs, CD11b^lo^Ly6C^lo^. Statistics is on the right (n=7). (C) Quantification of Ki67^+^ cells in CD45^+^F4/80^+^ liver cells and KCs from *Lm*-infected mice at 3 dpi. n=8. (D) Representative histogram of ZsGreen fluorescence in KCs isolated from TMEM16F-ZsGreen reporter mice (fluorescent intensity arbitrarily defined as low, intermediate, and high) (left) and representative histogram of DAPI staining in KCs with low, intermediate, and high level of ZsGreen after *Lm* infection (middle). The correlation analysis between ZsGreen and DAPI is on the right. n=12. (E) Flow cytometry of ZsGreen in splenic macrophages (CD11b^+^F4/80^+^), monocytes (CD11b^+^Gr-1^+^Ly6C^+^), and neutrophils (CD11b^+^Ly6G^+^). Each dot represents individual mouse in A to C. The “n” represents the technical replicates in D. Statistical analysis by two-tailed Mann-Whitney test (A), unpaired Student’s *t* test (B and C) or Spearman correlation analysis for D. ns, not significant. *p< 0.05, **p< 0.01.


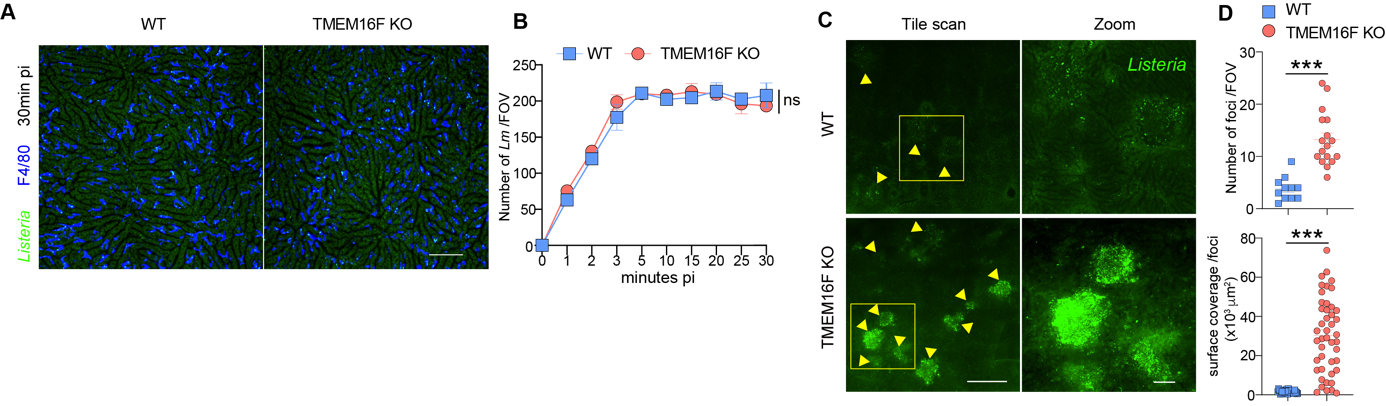


**Fig. S9. Monitoring *Lm* infection *in vivo* by intravital microscopy.** (A) Representative images of bacteria captured by KCs in WT and TMEM16F KO mice after the GFP-expressing *Lm* infection for 30 mins. Scale bar, 100 μm. (B) Quantification of the GFP-expressing *Lm* in liver immediately after bacteria injection in (A) (n=3). (C) As in (A), but mice were infected with 1x10^6^ GFP-expressing *Lm* for 48 hours. Scale bar, 500 μm in tile scan and 100 μm in zoom. (D) Quantification of number of foci per field of view (FOV) (n=11-17) and surface coverage per foci (n=34-45) after GFP-expressing *Lm* infection in (C). All the experiments were repeated for at least twice independently. Data are pooled from at least 2 independent experiments and presented as mean ± SEM. Statistical analysis by two-way ANOVA with Dunnett’s multiple comparisons test (B) or two-tailed Mann-Whitney test (D). ns, not significant; ***p< 0.001.

**Video S1.** Kupffer cell fragmentation by *Lm* infection.

**Video S2.** Loss of the plasma membrane integrity in Kupffer cells upon *Lm* infection
